# Measuring conductance switching in single proteins using quantum tunneling

**DOI:** 10.1126/sciadv.abm8149

**Published:** 2022-05-18

**Authors:** Longhua Tang, Long Yi, Tao Jiang, Ren Ren, Binoy Paulose Nadappuram, Bintian Zhang, Jian Wu, Xu Liu, Stuart Lindsay, Joshua B. Edel, Aleksandar P. Ivanov

**Affiliations:** 1 State Key Laboratory of Modern Optical Instrumentation, College of Optical Science and Engineering, International Research Center for Advanced Photonics, Zhejiang University, Hangzhou 310027, China.; 2 Innovation Institute for Artificial Intelligence in Medicine, Zhejiang-California International NanoSystems Institute, Zhejiang University, Hangzhou 310000, China.; 3 Department of Chemistry, Molecular Science Research Hub, Imperial College London, White City Campus, 82 Wood Lane, London W12 0BZ, UK.; 4 Department of Pure and Applied Chemistry, University of Strathclyde, Technology and Innovation Centre, 99 George Street, Glasgow G1 1RD, UK.; 5 Biodesign Institute; School of Life Sciences; Department of Physics; School of Molecular Sciences, Arizona State University, Tempe, AZ 85287, USA.

## Abstract

Interpreting the electrical signatures of single proteins in electronic junctions has facilitated a better understanding of the intrinsic properties of proteins that are fundamental to chemical and biological processes. Often, this information is not accessible using ensemble and even single-molecule approaches. In addition, the fabrication of nanoscale single-protein junctions remains challenging as they often require sophisticated methods. We report on the fabrication of tunneling probes, direct measurement, and active control (switching) of single-protein conductance with an external field in solution. The probes allowed us to bridge a single streptavidin molecule to two independently addressable, biotin-terminated electrodes and measure single-protein tunneling response over long periods. We show that charge transport through the protein has multiple conductive pathways that depend on the magnitude of the applied bias. These findings open the door for the reliable fabrication of protein-based junctions and can enable their use in future protein-embedded bioelectronics applications.

## Teaser

Single-protein tunneling junctions are used to study dynamic conductance switching of single proteins by external voltage bias.

## INTRODUCTION

The goal of molecular bioelectronics is to design nanoscale platforms that allow electronic signal transduction through/within biological molecules. Proteins are the key building blocks in biology and have remarkably versatile molecular recognition capability. The integration of proteins into bioelectronic devices has been a long-sought-after goal with a plethora of bioelectronic and biosensing applications (*1*–*6*). Especially, understanding and manipulation of protein-mediated electronic charge transport are of fundamental importance in electrochemical processes, in tunneling detection, and ultimately in the rational design of the next generation of bioelectronic devices (*1*, *2*, *7*–*11*).

Typically, protein-mediated tunneling electrical junctions rely on capturing proteins between a pair of closely spaced electrodes with a sub-5-nm gap. In such a configuration, it is possible to measure the tunneling current passing through the junction and the molecule as a function of the applied voltage bias (*2*, *5*, *9*). The characteristics of electronic charge transport are influenced by the protein structure, electrode-protein interface, and alignment of the energy levels, all of which affect the junction performance. Earlier studies demonstrated the feasibility of redox-active proteins to work as active components in micro/nanoscale molecular electronic circuits, providing the capability to control the charge transport through the protein entities and to induce electronic functions (*1*–*6*). In particular, the metalloproteins, such as Cu-azurin and cytochrome c, have been studied at the single-protein level, as they offer tunable redox properties and distinctive electrical response (*9*, *12*–*14*), whereas nonredox proteins are expected to be insulators (*1*, *2*). Recently, pioneering work has demonstrated that several nonredox proteins can also be conductive so long as charges could be injected into their interior via ligands or other physiochemical contacts (*1*, *10*, *15*, *16*). These findings suggest that nonredox proteins could also be potential candidates for biomolecular tunneling electronics, although the exact transport mechanism remains unclear.

Single-protein–coupled quantum mechanical tunneling (QMT) probes that allow the capture and analysis of individual proteins in a fixed tunneling gap could provide a powerful way to measure protein conformation in real time, enabling new types of single-molecule sensors and even sequencing platforms (*5*, *12*–*15*, *17*–*21*). In recent years, much effort has been expended on the development of methods for the fabrication of stable QMT probes, with the most commonly used methods deploying proximal probe technology, such as scanning probe microscopy (*1*, *16*, *22*), mechanically formed break junctions (*23*), and lithographically formed nanogaps (*10*, *14*, *24*). Each method has its advantages, with scanning tunneling microscopy break junctions (STM-BJ) offering high spatial control and variable gap distance, while lithography-based methods, such as mechanically controllable break junctions, allow fabrication on a larger scale (*23*, *25*–*27*). However, it is often difficult to embed proteins in a fixed gap using state-of-the-art methods (*1*, *16*, *28*). For instance, the reproducibility and stability of single-protein junctions remain challenging; as a result, most of the methods used predominantly rely on using a monolayer of redox-active proteins in an essentially dry state, and few have been investigated in solution (*1*, *2*, *4*). Hence, effective methods to study the electronic/electrochemical characteristics of individual proteins over long periods are essential to further develop the field of protein-embedded tunneling bioelectronics.

**Fig. 1.**
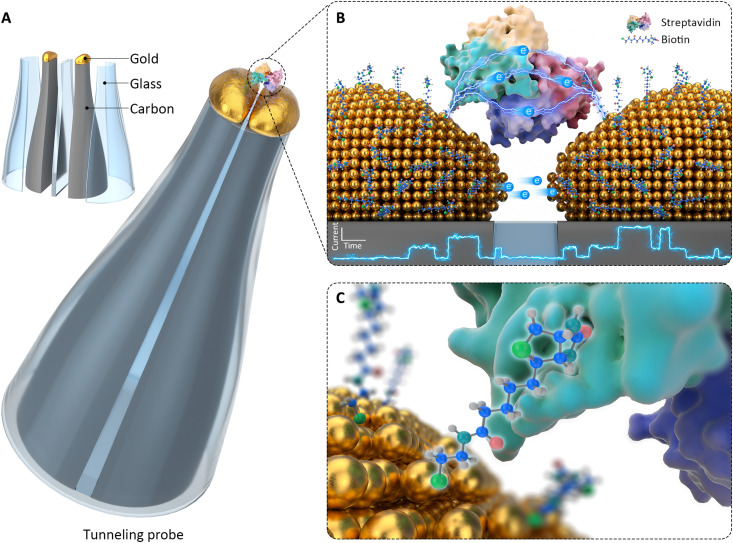
Schematic of a single streptavidin protein-coupled QMT probe. (**A**) The probe consists of two carbon electrodes that are sealed in a double-barrel quartz nanopipette. Only the tip of the electrode surface at the nanopipette tip is exposed to the solution. Gold is electrochemically deposited on the carbon nanoelectrodes using real-time current feedback, resulting in the formation of a tunneling junction with a controllable conductance. (**B**, **C**) The stand-alone QMT probe was functionalized with thiolated biotin and used to capture individual streptavidin molecules.

We recently reported on the fabrication and validation of stand-alone QMT probes consisting of a controllable and stable gap that can be used directly in solution (*29*). Here, we demonstrate protein-coupled QMT of single nonredox active proteins in solution. We used the nonredox active tetrameric streptavidin, which offered four binding sites to biotin. The tunneling electrode surface was functionalized with thiolated biotin, and the biotin on each QMT nanoelectrode could be used as an anchor to bind to the streptavidin (Fig. 1). Using this configuration, it was possible to record stable conductance time traces over a long duration (up to 2 hours). The probe stability is remarkable and allows the direct observation and active control (switching) of single-protein conductance with an external field.

## RESULTS

### Tunneling probe fabrication and functionalization

**Fig. 2.**
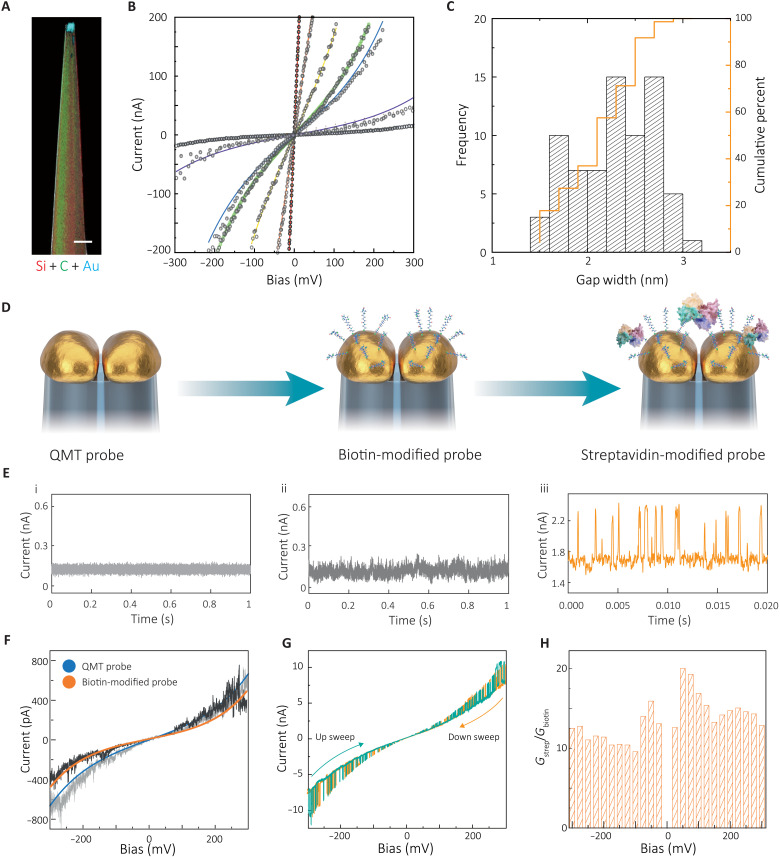
Schematic representation and electrical characterization of the streptavidin-coupled QMT probes. (**A**) STEM-EDX mapping of a QMT probe tip confirming silicon, deposited carbon, and electrodeposited gold. Scale bar, 50 nm. (**B**) Tunneling current-bias (*I*-*V*) plot of QMT probes in air at room temperature (297 K). The solid curves represent fits based on the Simmons tunneling model. (**C**) Histogram of the gap distance as obtained from the Simmons model for QMT probes measured in air. (**D**) Schematic representation of the QMT probes used. (**E**) Chronoamperometric (*I*-*t*) measurements for QMT probe (i) before and (ii) after modification with biotin and (iii) single streptavidin protein-coupled probe in 1 mM PBS at room temperature (297 K). Individual current transients are visible in streptavidin-bound junctions. Bias, 100 mV. (**F**) Current-bias plot for QMT probe before and after modification with biotin. The solid curves represent fits based on the Simmons tunneling model. (**G**) *I*-*V* measurement of the streptavidin-bridged QMT probes by sweeping the bias between −300 and +300 mV. Similar to (E) (iii), individual current transients are visible in streptavidin-bound junctions. (**H**) Ratio at a different bias of the baseline conductance measured in streptavidin-bound junctions (*G*_strep_) and the conductance (*G*_biotin_) of biotin-modified QMT probe.

Figure 1 illustrates the probe design and architecture. The QMT probe consists of two individually addressable nanoelectrodes in a stand-alone double-barrel nanopipette (*29*). Briefly, theta-shaped double-barreled quartz capillaries were laser-pulled to a sharp tip with two nanopores spaced by a quartz septum of 10 to 20 nm. Next, the barrels were filled with butane gas to deposit conductive carbon via pyrolysis. This results in two semielliptical coplanar nanoelectrodes at the tip of the pipette. Subsequently, gold was electrochemically deposited using real-time tunneling feedback control to tune the gap distance. Characterization of the barrels and the tunneling junctions via optical images and scanning transmission electron microscopy (STEM)–energy-dispersive x-ray spectrometry (EDX) revealed that gold was deposited at the tips of the probes (Fig. 2A and fig. S1). Last, the QMT probes were thoroughly cleaned by ethanol and deionized water and then functionalized with thiolated biotin in ethanol, yielding the biotin-modified QMT probes.

To demonstrate the functionality of the QMT probes, tunneling current-bias (*I*-*V*) measurements were conducted in air at room temperature (297 K). The probes had a typical nonlinear current-bias response and were fitted to the Simmons tunneling model, which was used to extract approximate parameters such as gap distance and barrier height (see Fig. 2B and fig. S2) (*30*). To maximize the signal-to-noise ratio (SNR) and the likelihood of bridging the QMT junction, electrode gaps ranging from 1.5 to 3.1 nm (as calculated using the Simmons model) were selected (Fig. 2C). The distance was larger than the size of thiolated biotin (molecular weight: 302 Da; size: <1 nm) but smaller than that of streptavidin (tetramer, 52.8 kDa; size: ~5 nm), allowing the streptavidin to bridge the gap by binding to single biotin on each electrode. It was not necessary for streptavidin to be fully confined in the gap, as the current tunneling through the biotin contacts and the protein dominated the conductance. Larger gaps (≥5 nm) that could accommodate the full protein are challenging to fabricate in a tunneling feedback regime and exhibit low SNR because of an exponential decrease in the magnitude tunneling current with gap distance.

The effective tunneling gap distances before and after modification of biotin were analyzed. The measured *I*-*V* curves for bare and biotin-modified QMT exhibited stable tunneling current-bias characteristics, consistent with the Simmons tunneling model (Fig. 2E). The gap distances were estimated to be 2.45 nm for bare QMT probes and 2.35 nm for biotin-modified probes, indicating a stable and functional tunneling gap during modification. After biotin functionalization, minor changes in the background current were observed (fig. S3). Long-term measurements confirmed that QMT probes with biotin functionalization were stable over 2 weeks when stored under ambient conditions (fig. S4).

### Molecular junction formation and characterization

The formation of a single protein junction was monitored in real time by measuring the tunneling current over time at an applied bias of 100 mV in 1 mM phosphate-buffered saline (PBS) at pH 7.4. Without proteins, a stable tunneling current was observed for the bare and biotin-modified QMT probes (Fig. 2E). When streptavidin (0.2 ng/ml) was added, an increase in the baseline tunneling current could be observed, followed by frequent transient events (see representative trace iii in Fig. 2E and fig. S5). A comparison of the power spectra density calculated for the same QMT probes before and after modification with single streptavidin revealed an increase in the low-frequency regime *f* < 10 Hz, where the power spectrum shows a characteristic 1/*f* dependence (fig. S6). This relative increase in 1/*f* noise may be attributed to streptavidin binding and dynamic fluctuations of the molecules in the QMT junction (*31*).

The electrical properties of protein junctions were investigated by *I*-*V* measurements, where the applied bias was continuously swept; see representative *I*-*V* curves in Fig. 2 (F and G). A typical sigmoidal *I*-*V* plot was observed in the bias range between −0.3 and +0.3 V at a sweep rate of 0.8 V s^−1^. Superimposed on the *I*-*V* curves bias, dependent current increase transients were also seen (Fig. 2G). This can be visualized more clearly by plotting the conductance as a function of bias (fig. S7). As the bias increased, the current transient had a greater magnitude. Similar large current transients (often termed as telegraph noise) have been observed for integrin and other proteins in modified STM probes (*1*, *15*). A conductance increase was highly reproducible between different junctions and for a range of biases (Fig. 2H). The measured conductance was in good agreement with values previously obtained for noncovalent streptavidin-biotin coupling linked to the electrodes via a thiol-terminated ethane linkage (*22*). In contrast, both bare and biotinylated junctions did not show significant current transients, indicating that the latter originate from the interaction of single streptavidin and the biotinylated tunneling junction (Fig. 2F). The cycled *I*-*V* sweeps, as shown in Fig. 2G, and the baseline of the *I*-*V* curves, including the amplitude of the current transients, were highly reproducible and overlapped over multiple repeats, indicating a relatively and good stability of protein-coupled QMT probe over the applied bias range. It is essential to operate in a low-bias regime (<|300 mV|), as a higher bias often resulted in a sudden drop in current, which can be attributed to a breakage of the biotin-streptavidin-biotin contacts (fig. S8).

We previously reported that nonredox proteins, including streptavidin, could be detected in bare QMT probes. In that case, the current transients were attributed to the protein diffusing in and out of the junction, and the magnitude of the signal could be used to differentiate between different proteins (*29*). Here, the addition of proteins that are not expected to specifically bind to biotin-modified QMT probes [immunoglobulin G, bovine serum albumin, and thrombin (0.2 ng/ml)] resulted in a minor change in the tunneling current, but with a substantial increase in baseline noise and with only very few current transients (fig. S9, A to D).

In addition, control experiments were performed with monovalent streptavidin (SAvPhire, Sigma-Aldrich) with only one available binding site. A current rise could be observed (fig. S9E); however, the distinct multilevel switching that was observed in streptavidin-bridged junctions was not evident with monovalent streptavidin.

Together, these results confirmed the specific interaction between streptavidin tetramer and the functionalized electrodes. Overall, approximately 31% of all resulted streptavidin-coupled probes (15 of 49) exhibited stable and reproducible current-bias behavior with reproducible current transients (see fig. S10).

**Fig. 3.**
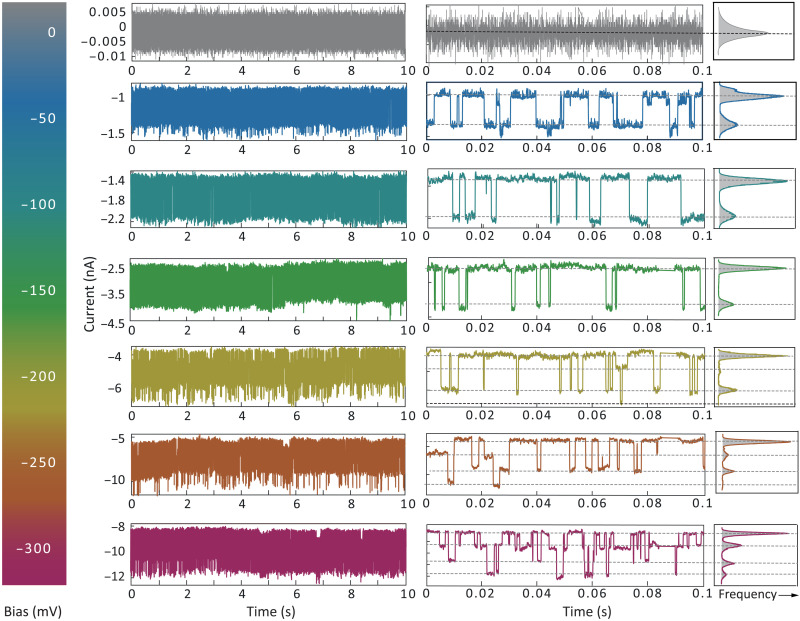
Chronoamperometric traces of single streptavidin-bridged QMT probes at different bias using 1 mM PBS (pH 7.4) at 298 K. Representative 10-s *I*-*t* traces are shown on the left, and zoomed-in 0.1-s traces are on the middle. The emergence of multiple conductance states becomes visible with increasing applied bias. The corresponding all-point current histograms are plotted on the right.

To further investigate the bias dependence on the protein conductance characteristics, we carried out real-time current measurements at the different bias conditions. Figure 3 presents typical current-time traces at the bias ranging between −300 and 0 mV, where pulse-like stochastic switching between the levels could clearly be seen. Similar to the long-time measurement at the different bias conditions, all the current signals exhibited consistent characteristics, demonstrating good stability of protein junction and reproducibile current switching (fig. S11). These traces were used to construct current histograms (right column in Fig. 3). When the negative bias was systematically increased in the range from −25 to −300 mV, the distribution of the current transients increased from two to up to four distinct current levels at a higher bias. We also carried out experiments at positive bias ≤+200 mV (fig. S12), and a response similar to the one at negative bias was observed (fig. S13).

### Statistical analyses of dynamic conductance switching

**Fig. 4.**
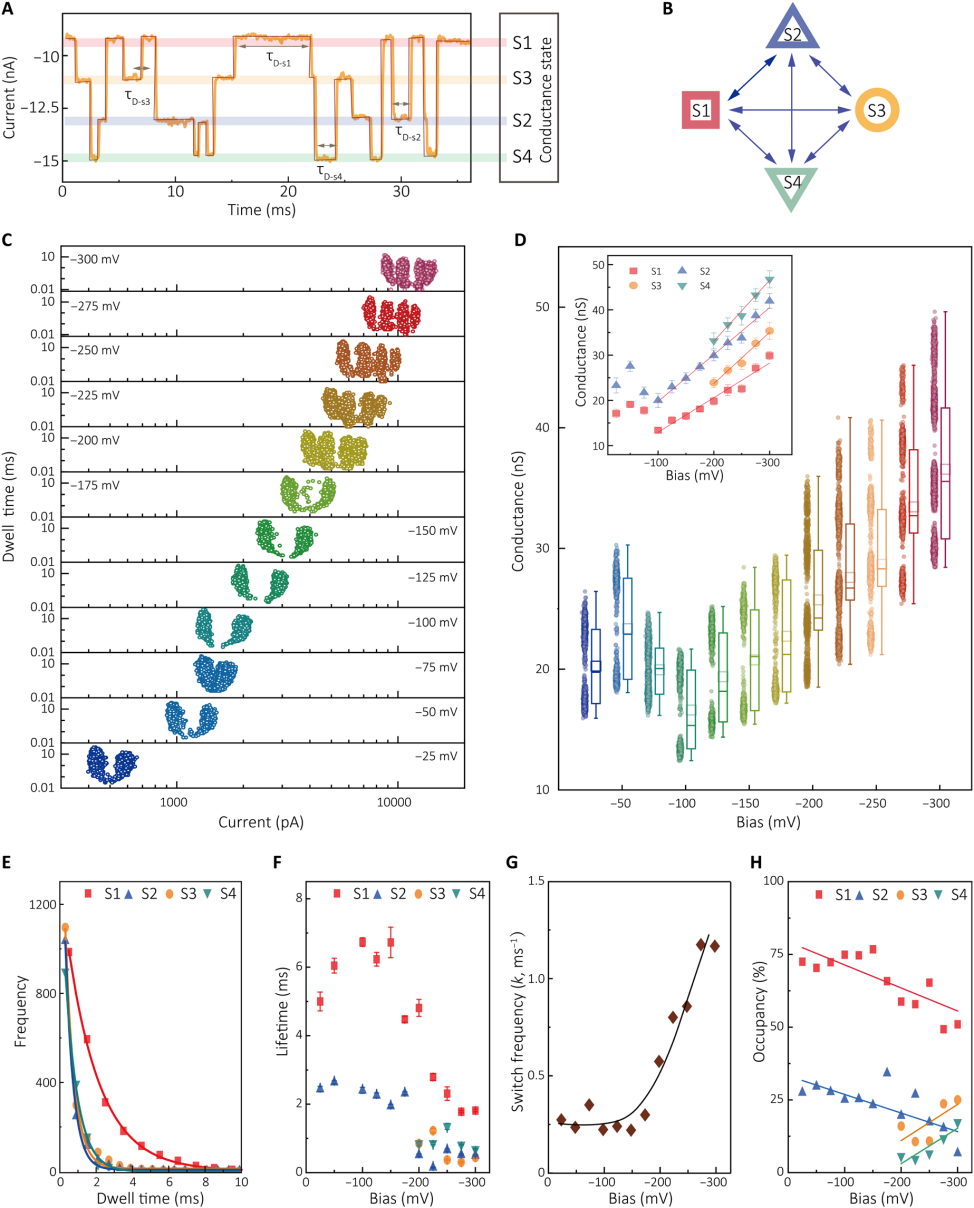
Statistical analysis of stochastic conductance switching for single streptavidin-coupled QMT probes. (**A**) *I*-*t* current trace segment recorded at −300 mV in PBS (pH 7.4) at 298 K (orange). Idealized fit obtained from a segmental *k*-means method based on hidden Markov model analysis using QUB (dark red). (**B**) Interconversion between each conductance state for the current-time trace in (A). (**C**) Scatterplots of dwell time versus current amplitude at different constant bias. (**D**) Combined conductance scatter and box plots at different constant bias. (Inset) Plot of each conductance state of streptavidin-coupled QMT probe as a function of bias. (**E**) Plots of the dwell time for each state at −300 mV using an idealized fit using QUB. The solid lines correspond to monoexponential fits from which the lifetime constants of the four states have been calculated. (**F**) Lifetime plot for each conductance state at a different constant bias. (**G**) Conductance switching rate constant *k* and occupancy (**H**) for each state at different bias. The resulting kinetic parameters are listed in table S1.

We used hidden Markov modeling and simulations [Quantify Unknown Biophysics (QUB); qub.mandelics.com] to perform idealized fitting of the *I*-*t* trajectories with multiple plain states that were derived from a segmental *k*-means method (*30*, *32*, *33*). An example is shown in Fig. 4A, where the representative *I*-*t* trace at −300 mV was idealized to quadruple conductance state interconversion (Fig. 4B); thereby, the stochastic conductance switching and the dwell time of each state were statistically analyzed. Together with the analyses of *I*-*t* curves at different bias, the corresponding current amplitude versus dwell time histogram of each bias was obtained (Fig. 4C and fig. S14). We found that, with increasing bias, the current markedly grew. The conductance and the current amplitude followed a Gaussian distribution (Fig. 4D and fig. S15). At negative bias ranging from −25 to −175 mV, two distinct conductance states, named S1 and S2, respectively, were observed. At −200 mV, two additional states, S3 and S4, appeared along with the initial S1 and S2 states. As the bias increased, the segmentation of these quadruple conductance states was more pronounced (Fig. 4D). Each state’s conductance increased linearly between −100 and −300 mV (Fig. 4D, inset).

As illustrated above, the current measurements for S1 resulted in a narrow distribution for all applied bias settings. The conductance peaks of S1 also agreed well with the *I*-*V* data in Fig. 2G (see fig. S16A). The current measured with streptavidin increased compared to junctions without streptavidin at the same bias setting. When biasing the streptavidin-bridged QMT probe, electrons would partially pass through the tunneling gap. The measured current in the streptavidin-bridged QMT probe combines the tunneling current through gap space and electron transport through protein molecules. Hence, the current contribution of the bound streptavidin can be estimated as the current difference between the biotin-modified and the streptavidin-bridged QMT probe. The protein conductance can also be estimated under different bias conditions (fig. S16B).

To gain more information on the conductance switching dynamics, the dwell time (*t*_D_) of each conductance state was further analyzed (*34*–*36*). Idealized fitting revealed a multiple-state interconversion. Figure 4E corresponds to the dwell time distribution of each state of a typical signal at an applied bias of −300 mV. The dwell time distribution for the quadruple conductance states exhibits a single exponential distribution. The dwell time distribution dependence on bias was also analyzed, and the mean lifetime of each state was obtained. The lifetime constants (τ) for the quadruple conductance states were τ_s1_ = 1.81 ± 0.03 ms, τ_s2_ = 0.50 ± 0.03 ms, τ_s3_ = 0.45 ± 0.02 ms, and τ_s4_ = 0.68 ± 0.02 ms, respectively (Fig. 4F, figs. S17 to S20, and table S1). We found that, when the bias magnitude was lower than −200 mV, longer lifetimes for the current state S1 (τ_s1_) and state S2 (τ_s2_) were induced than the lifetimes obtained at higher bias. τ_s1_ reached a maximum at around −100 mV (6.74 ± 0.13 ms) and then decreased significantly (5.00 ± 0.27 ms at −25 mV and 4.81 ± 0.25 ms at −200 mV). In contrast, τ_s2_ remained at ~2 ms at a bias of <200 mV while decreasing significantly from −200 to −300 mV. This corresponds to the reduced energy barrier for state S1 at higher bias, leading to the acceleration of the switching rate. At the same time, the mean values for τ_s3_ and τ_s4_ for current state S3 and state S4, respectively, remained ~1 ms between −200 and −300 mV. The conductance switching rate constant, *k* = 1/τ, can be obtained from a maximum likelihood estimate of the lifetime at a different bias (Fig. 4G). Analysis of the transition probabilities for each conductance state at different bias indicates that the transition S1 ↔ S2 is most probable, followed by S2 ↔ S3, with S2 ↔ S4 being least probable in the regime (−200 to −300 mV) (see figs. S21 to S26 and table S2).

Because at least two conductance states were observed at each bias setting, two or more electron transport pathways may exist for the protein-coupled tunneling junction. However, each bias shows different amounts of current states, revealing that there are likely undetected or inactivated states at low bias. For example, S3 and S4 pathways existed at −175 mV but were scarcely observed (<1% of the total transitions; see fig. S11 and Fig. 4C), indicating that conductance S1 and S2 dominated the total junction conductance. A possible explanation is that the occurrence frequency of the corresponding states is too low to be detected. The occurrence of each state was dependence on the bias conditions (Fig. 4H). As the bias magnitude increased from −25 to −300 mV, the occupancy for conductance states of S1 and S2 decreased from 72.5 to 51.0% and 27.5 to 6.7%, respectively whereas, the occupancy for states S3 and S4 increased.

## DISCUSSION

We observed that the biotin-streptavidin-biotin protein junction had a relatively high conductance in the order of tens of nanosiemens over a wide bias range and exhibited stochastic switching behavior among different conductance states. Compared to previous reports (*23*, *30*), our single-protein–modified QMT probes exhibited higher conductance over a wide bias range (−300 to 200 mV) and enabled the electrical measurement of a single molecule over long periods, which can benefit the study of protein conformation and function using tunneling spectroscopy. In the presence of streptavidin tetramer, a significantly higher current was passed through the tunneling junction. As the bias increased, the conductance of the observed states grew linearly with bias. However, the conductance ratio *G*_strep_/*G*_biotin_ remained largely unchanged at a different bias (Fig. 2H), indicating the availability of a non-ohmic conductance channel when current transients and conductance switching is observed. The detectable bias threshold for conductance switching for streptavidin was observed to be at 25 mV or higher. This may be attributed to several factors, including the better junction stability, lower leakage current due to improved electrode design, and the relatively narrow fixed-gap junction with an averaged gap distance of 2.3 ± 0.4 nm. Streptavidin is a tetrameric protein, and the four individual conductance states observed may indicate a possible change of confirmation and potentially structural switching that corresponds to a change in conductance. A confirmation of this requires follow-up measurements at a higher applied bias that will reveal whether there are other conductance states present. Currently, the protein junctions are not sufficiently stable in the high-bias regime to investigate this systematically, but measurements can be attempted in the future with improved probe design and optimized ligand chemistry.

Although the exact mechanism of the protein conductance switching by an external field is still subject to discussion, some conclusions can be reached from observations and statistical analysis. Because the streptavidin protein was specifically bonded to the QMT probe by the two biotin anchoring sites, the biotin-streptavidin interactions could only contact streptavidin at a certain angle, thus reducing the possibility of the protein stretching and conformation rotation. Moreover, the conductance might be related to the molecular polarity in the strong electric field (*14*, *17*, *37*). When the proteins are in solution under a higher electric field, the dipole moment of the protein may increase, thereby leading to the enhancement of the interaction between electrode and protein (*7*, *33*, *35*). Such behavior may be described within both the polaron and the charge hopping models. Recently, it has been suggested that resonant tunneling (represented by the Landauer-Büttiker formalism) may be the dominant conduction mechanism in nonredox protein junctions (*1*, *23*, *38*). In both the hopping and the resonant tunneling model, bias-dependent stochastic switching characteristics may be driven by the transient molecular charging, possibly because of protein conformational change under the electric field. The current-bias (*I*-*V*) characteristics reported in Fig. 3 and figs. S12 and S13 are consistent with this model if the multiple locally stable conformations have different conducting capability. When the states are of comparable stability and the barrier between them, transitions can manifest as stochastic fluctuations in the system conductance (*16*, *39*). Reliable discrimination between the hopping and resonant tunneling mechanisms for conduction requires further experimental and theoretical analysis. In either mechanism, the energy gap between the highest occupied molecular orbital and the gold Fermi level plays a crucial role in determining the threshold bias for conduction (*10*). Therefore, to elucidate the underlying transport mechanism leading to the observed bias dependence, measurements at low temperatures are likely needed (*13*, *18*, *40*).

In conclusion, we were able to actively control switching of single-protein conductance with an external field and to study stochastic fluctuations under a solution-based environment at room temperature. The probes presented here can offer a viable route to access conductance pathways of individual protein molecules and their interaction with ligands. This may provide valuable information on conformational states related to biological processes and offer novel modes of protein biosensing and bioelectronics.

## MATERIALS AND METHODS

### Materials

Wild-type streptavidin tetramer was purchased from Sigma-Aldrich. The biotin disulfides were synthesized according to a previous report (*22*) and treated by the immobilized (tris[2-carboxyethyl]phosphine hydrochloride) disulfide reducing gel from Thermo Fisher Scientific (catalog number 77712) following the manufacturer’s instructions. All other chemicals used in the probe fabrication and electrochemical characterization were bought from Sigma-Aldrich, apart from the plating solution. The plating solution was obtained from Tianyue (China), which has 5 g in 250 ml of electrolyte.

### Fabrication of QMT probes

The QMT probes were fabricated from theta-shaped dual-barrel quartz capillaries via our recently reported method (*29*). Briefly, quartz capillaries (Friedrich & Dimmock Inc.; 1.2 mm outside diameter, 0.90 mm inside diameter, 100 mm long) were pulled to sharp tips using a P-2000 laser puller (Sutter Instrument). Both barrels were then filled inside by carbon pyrolytic decomposition of butane, resulting in the two coplanar semielliptical carbon nanoelectrodes separated by a quartz septum. Next, gold was electrochemically deposited onto the carbon nanoelectrodes with a feedback-controlled chronopotentiometric step to decrease the electrode gap within the QMT probe regime. Last, stable QMT nanoprobes were immersed in ultrapure deionized water (with a resistivity of 18.2 megohm·cm) for 12 to 48 hours. The functionality of the QMT probe junctions was characterized experimentally by recording *I*-*V* curves and fitting the data to the Simmons model, which can be used to approximate the gap distance, tunneling barrier height, and tunneling area.

### Preparation of streptavidin QMT probes

A detailed description of the preparation of the streptavidin QMT probes is shown in fig. S28. Briefly, the QMT probes were cleaned using deionized water and ethanol and then immersed in ethanol containing 100 μM thiolated biotin overnight. We used the *N*,*N*′-bisbiotinyl cystamine for biotin modification (fig. S28). Next, the biotin-modified QMT probes were taken out from the solution, washed with abundant deionized water to remove excess biotin and ethanol, and used immediately. The tunneling current for the biotin-QMT probes was continuously recorded at 50 mV in 1 mM PBS (pH 7.4) containing streptavidin (0.2 ng/ml). Once two-level transients were detected, the QMT nanoprobes were rinsed in 1 mM PBS.

### Electrical measurements

All tunneling current measurements were done using MultiClamp 700B operated in voltage-clamp mode. The data acquisition bandwidth was 100 kHz, and the data were filtered using a four-pole Bessel filter at 10 kHz and digitized using Axon Digidata 1550B. Data analysis was performed using a custom-written MATLAB code developed in-house and also QUB software.

## Notes

View/request a protocol for this paper from *Bio-protocol*.

## References

[1] S. Lindsay, Ubiquitous electron transport in non-electron transfer proteins. Life 10, 72 (2020). 32443721 10.3390/life10050072 PMC7281237

[2] C. D. Bostick, S. Mukhopadhyay, I. Pecht, M. Sheves, D. Cahen, D. Lederman, Protein bioelectronics: A review of what we do and do not know. Rep. Prog. Phys. 81, 026601 (2018). 29303117 10.1088/1361-6633/aa85f2

[3] D. Xiang, X. Wang, C. Jia, T. Lee, X. Guo, Molecular-scale electronics: From concept to function. Chem. Rev. 116, 4318–4440 (2016). 26979510 10.1021/acs.chemrev.5b00680

[4] A. Vilan, D. Aswal, D. Cahen, Large-area, ensemble molecular electronics: Motivation and challenges. Chem. Rev. 117, 4248–4286 (2017). 28177226 10.1021/acs.chemrev.6b00595

[5] N. Amdursky, D. Marchak, L. Sepunaru, I. Pecht, M. Sheves, D. Cahen, Electronic transport via proteins. Adv. Mater. 26, 7142–7161 (2014). 25256438 10.1002/adma.201402304

[6] M. Di Ventra, M. Taniguchi, Decoding DNA, RNA and peptides with quantum tunnelling. Nat. Nanotechnol. 11, 117–126 (2016). 26839257 10.1038/nnano.2015.320

[7] N. Xin, J. Guan, C. Zhou, X. Chen, C. Gu, Y. Li, M. A. Ratner, A. Nitzan, J. F. Stoddart, X. Guo, Concepts in the design and engineering of single-molecule electronic devices. Nat. Rev. Phys. 1, 211–230 (2019).

[8] J. A. Fereiro, X. Yu, I. Pecht, M. Sheves, J. C. Cuevas, D. Cahen, Tunneling explains efficient electron transport via protein junctions. Proc. Natl. Acad. Sci. U.S.A. 115, E4577–E4583 (2018). 29712853 10.1073/pnas.1719867115 PMC5960296

[9] J. A. Fereiro, G. Porat, T. Bendikov, I. Pecht, M. Sheves, D. Cahen, Protein electronics: Chemical modulation of contacts control energy level alignment in gold-azurin-gold junctions. J. Am. Chem. Soc. 140, 13317–13326 (2018). 30235415 10.1021/jacs.8b07742

[10] B. Zhang, S. Lindsay, Electronic decay length in a protein molecule. Nano Lett. 19, 4017–4022 (2019). 31144824 10.1021/acs.nanolett.9b01254 PMC7147071

[11] B. Zhang, E. Ryan, X. Wang, S. Lindsay, Probing bioelectronic connections using streptavidin molecules with modified valency. J. Am. Chem. Soc. 143, 15139–15144 (2021). 34499834 10.1021/jacs.1c05569 PMC8458255

[12] J. M. Artés, I. Díez-Pérez, P. Gorostiza, Transistor-like behavior of single metalloprotein junctions. Nano Lett. 12, 2679–2684 (2012). 21973084 10.1021/nl2028969

[13] L. Sepunaru, N. Friedman, I. Pecht, M. Sheves, D. Cahen, Temperature-dependent solid-state electron transport through bacteriorhodopsin: Experimental evidence for multiple transport paths through proteins. J. Am. Chem. Soc. 134, 4169–4176 (2012). 22296717 10.1021/ja2097139

[14] J. A. Fereiro, T. Bendikov, I. Pecht, M. Sheves, D. Cahen, Protein binding and orientation matter: Bias-induced conductance switching in a mutated azurin junction. J. Am. Chem. Soc. 142, 19217–19225 (2020). 33141577 10.1021/jacs.0c08836 PMC7662909

[15] B. Zhang, W. Song, P. Pang, Y. Zhao, P. Zhang, I. Csabai, G. Vattay, S. Lindsay, Observation of giant conductance fluctuations in a protein. Nano Futures 1, 035002 (2017). 29552645 10.1088/2399-1984/aa8f91 PMC5851656

[16] B. Zhang, W. Song, J. Brown, R. Nemanich, S. Lindsay, Electronic conductance resonance in non-redox-active proteins. J. Am. Chem. Soc. 142, 6432–6438 (2020). 32176496 10.1021/jacs.0c01805 PMC7185870

[17] J. A. Fereiro, B. Kayser, C. Romero Muñiz, A. Vilan, D. A. Dolgikh, R. V. Chertkova, J. C. Cuevas, L. A. Zotti, I. Pecht, M. Sheves, D. Cahen, A solid-state protein junction serves as a bias-induced current switch. Angew. Chem. Int. Ed. 58, 11852–11859 (2019). 10.1002/anie.201906032 31246354

[18] B. Kayser, J. A. Fereiro, R. Bhattacharyya, S. R. Cohen, A. Vilan, I. Pecht, M. Sheves, D. Cahen, Solid-state electron transport via the protein azurin is temperature-independent down to 4 K. J. Phys. Chem. Lett. 11, 144–151 (2020). 31821001 10.1021/acs.jpclett.9b03120

[19] O. E. Castañeda Ocampo, P. Gordiichuk, S. Catarci, D. A. Gautier, A. Herrmann, R. C. Chiechi, Mechanism of orientation-dependent asymmetric charge transport in tunneling junctions comprising photosystem I. J. Am. Chem. Soc. 137, 8419–8427 (2015). 26057523 10.1021/jacs.5b01241 PMC4558993

[20] G. Mezei, Z. Balogh, A. Magyarkuti, A. Halbritter, Voltage-controlled binary conductance switching in gold–4,4′-bipyridine–gold single-molecule nanowires. J. Phys. Chem. Lett. 11, 8053–8059 (2020). 32893638 10.1021/acs.jpclett.0c02185 PMC7528405

[21] B. Zhang, H. Deng, S. Mukherjee, W. Song, X. Wang, S. Lindsay, Engineering an enzyme for direct electrical monitoring of activity. ACS Nano 14, 1360–1368 (2020). 31594304 10.1021/acsnano.9b06875 PMC7047563

[22] B. Zhang, W. Song, P. Pang, H. Lai, Q. Chen, P. Zhang, S. Lindsay, Role of contacts in long-range protein conductance. Proc. Natl. Acad. Sci. U.S.A. 116, 5886–5891 (2019). 30846548 10.1073/pnas.1819674116 PMC6442609

[23] X. Zhuang, A. Zhang, S. Qiu, C. Tang, S. Zhao, H. Li, Y. Zhang, Y. Wang, B. Wang, B. Fang, W. Hong, Coenzyme coupling boosts charge transport through single bioactive enzyme junctions. iScience 23, 101001 (2020). 32259671 10.1016/j.isci.2020.101001 PMC7136626

[24] N. S. Hatzakis, L. Wei, S. K. Jorgensen, A. H. Kunding, P. Bolinger, N. Ehrlich, I. Makarov, M. Skjot, A. Svendsen, P. Hedegård, D. Stamou, Single enzyme studies reveal the existence of discrete functional states for monomeric enzymes and how they are "selected" upon allosteric regulation. J. Am. Chem. Soc. 134, 9296–9302 (2012). 22489643 10.1021/ja3011429

[25] J. Liu, X. Zhao, J. Zheng, X. Huang, Y. Tang, F. Wang, R. Li, J. Pi, C. Huang, L. Wang, Y. Yang, J. Shi, B. Mao, Z.-Q. Tian, M. R. Bryce, W. Hong, Transition from tunneling leakage current to molecular tunneling in single-molecule junctions. Chem 5, 390–401 (2019).

[26] J. Bai, A. Daaoub, S. Sangtarash, X. Li, Y. Tang, Q. Zou, H. Sadeghi, S. Liu, X. Huang, Z. Tan, J. Liu, Y. Yang, J. Shi, G. Mészáros, W. Chen, C. Lambert, W. Hong, Anti-resonance features of destructive quantum interference in single-molecule thiophene junctions achieved by electrochemical gating. Nat. Mater. 18, 364–369 (2019). 30742083 10.1038/s41563-018-0265-4

[27] L. Xiang, J. L. Palma, Y. Li, V. Mujica, M. A. Ratner, N. Tao, Gate-controlled conductance switching in DNA. Nat. Commun. 8, 14471 (2017). 28218275 10.1038/ncomms14471 PMC5321735

[28] M. P. Ruiz, A. C. Aragonès, N. Camarero, J. G. Vilhena, M. Ortega, L. A. Zotti, R. Pérez, J. C. Cuevas, P. Gorostiza, I. Díez-Pérez, Bioengineering a single-protein junction. J. Am. Chem. Soc. 139, 15337–15346 (2017). 28981262 10.1021/jacs.7b06130

[29] L. Tang, B. P. Nadappuram, P. Cadinu, Z. Zhao, L. Xue, L. Yi, R. Ren, J. Wang, A. P. Ivanov, J. B. Edel, Combined quantum tunnelling and dielectrophoretic trapping for molecular analysis at ultra-low analyte concentrations. Nat. Commun. 12, 913 (2021). 33568635 10.1038/s41467-021-21101-x PMC7876030

[30] J. G. Simmons, Generalized formula for the electric tunnel effect between similar electrodes separated by a thin insulating film. J. Appl. Phys. 34, 1793–1803 (1963).

[31] A. Fragasso, S. Pud, C. Dekker, 1/F noise in solid-state nanopores is governed by access and surface regions. Nanotechnology 30, 395202 (2019). 31247592 10.1088/1361-6528/ab2d35

[32] J. Guan, C. Jia, Y. Li, Z. Liu, J. Wang, Z. Yang, C. Gu, D. Su, K. N. Houk, D. Zhang, X. Guo, Direct single-molecule dynamic detection of chemical reactions. Sci. Adv. 4, eaar2177 (2018). 29487914 10.1126/sciadv.aar2177 PMC5817934

[33] C. Jia, A. Migliore, N. Xin, S. Huang, J. Wang, Q. Yang, S. Wang, H. Chen, D. Wang, B. Feng, Z. Liu, G. Zhang, D. H. Qu, H. Tian, M. A. Ratner, H. Q. Xu, A. Nitzan, X. Guo, Covalently bonded single-molecule junctions with stable and reversible photoswitched conductivity. Science 352, 1443–1445 (2016). 27313042 10.1126/science.aaf6298

[34] Z. Liu, X. Li, H. Masai, X. Huang, S. Tsuda, J. Terao, J. Yang, X. Guo, A single-molecule electrical approach for amino acid detection and chirality recognition. Sci. Adv. 7, abe4365 (2021). 10.1126/sciadv.abe4365 PMC7929498 33658198

[35] C. Yang, Z. Liu, Y. Li, S. Zhou, C. Lu, Y. Guo, M. Ramirez, Q. Zhang, Y. Li, Z. Liu, K. N. Houk, D. Zhang, X. Guo, Electric field-catalyzed single-molecule diels-alder reaction dynamics. Sci. Adv. 7, eabf0689 (2021). 33523936 10.1126/sciadv.abf0689 PMC7817103

[36] P. Li, C. Jia, X. Guo, Structural transition dynamics in carbonelectrode-based single-molecule junctions. Chin. J. Chem. 39, 223–231 (2021).

[37] E. Leary, B. Limburg, A. Alanazy, S. Sangtarash, I. Grace, K. Swada, L. J. Esdaile, M. Noori, M. T. González, G. Rubio-Bollinger, H. Sadeghi, A. Hodgson, N. Agraït, S. J. Higgins, C. J. Lambert, H. L. Anderson, R. J. Nichols, Bias-driven conductance increase with length in porphyrin tapes. J. Am. Chem. Soc. 140, 12877–12883 (2018). 30207150 10.1021/jacs.8b06338

[38] Y. Han, C. Nickle, Z. Zhang, H. P. A. G. Astier, T. J. Duffin, D. Qi, Z. Wang, E. Del Barco, D. Thompson, C. A. Nijhuis, Electric-field-driven dual-functional molecular switches in tunnel junctions. Nat. Mater. 19, 843–848 (2020). 32483243 10.1038/s41563-020-0697-5

[39] F. Schwarz, G. Kastlunger, F. Lissel, C. Egler-Lucas, S. N. Semenov, K. Venkatesan, H. Berke, R. Stadler, E. Lörtscher, Field-induced conductance switching by charge-state alternation in organometallic single-molecule junctions. Nat. Nanotechnol. 11, 170–176 (2016). 26571004 10.1038/nnano.2015.255

[40] S. Mukhopadhyay, S. Dutta, I. Pecht, M. Sheves, D. Cahen, Conjugated cofactor enables efficient temperature-independent electronic transport across ∼6 Nm long halorhodopsin. J. Am. Chem. Soc. 137, 11226–11229 (2015). 26301971 10.1021/jacs.5b06501

