# Supplementary material for: Measuring conductance switching in single proteins using quantum tunneling
Source: Sci Adv. 2022 May 18;8(20):eabm8149. doi: 10.1126/sciadv.abm8149 (PMC9116604; doi:10.1126/sciadv.abm8149)
Supplement: Supplementary file 1 — Figs. S1 to S28 Tables S1 and S2 [file sciadv.abm8149_sm.pdf]

Science Advances
AAAS

Supplementary Materials for

Measuring conductance switching in single proteins using quantum tunneling

Longhua Tang*, Long Yi, Tao Jiang, Ren Ren, Binoy Paulose Nadappuram, Bintian Zhang, Jian Wu, Xu Liu, Stuart Lindsay, Joshua B. Edel*, Aleksandar P. Ivanov*

*Corresponding author. Email: lhtang@zju.edu.cn (L.T.); alex.ivanov@imperial.ac.uk (A.P.I.); joshua.edel@imperial.ac.uk (J.B.E.)

Published 18 May 2022, *Sci. Adv.* **8**, eabm8149 (2022)
DOI: 10.1126/sciadv.abm8149

This PDF file includes:

Figs. S1 to S28
Tables S1 and S2

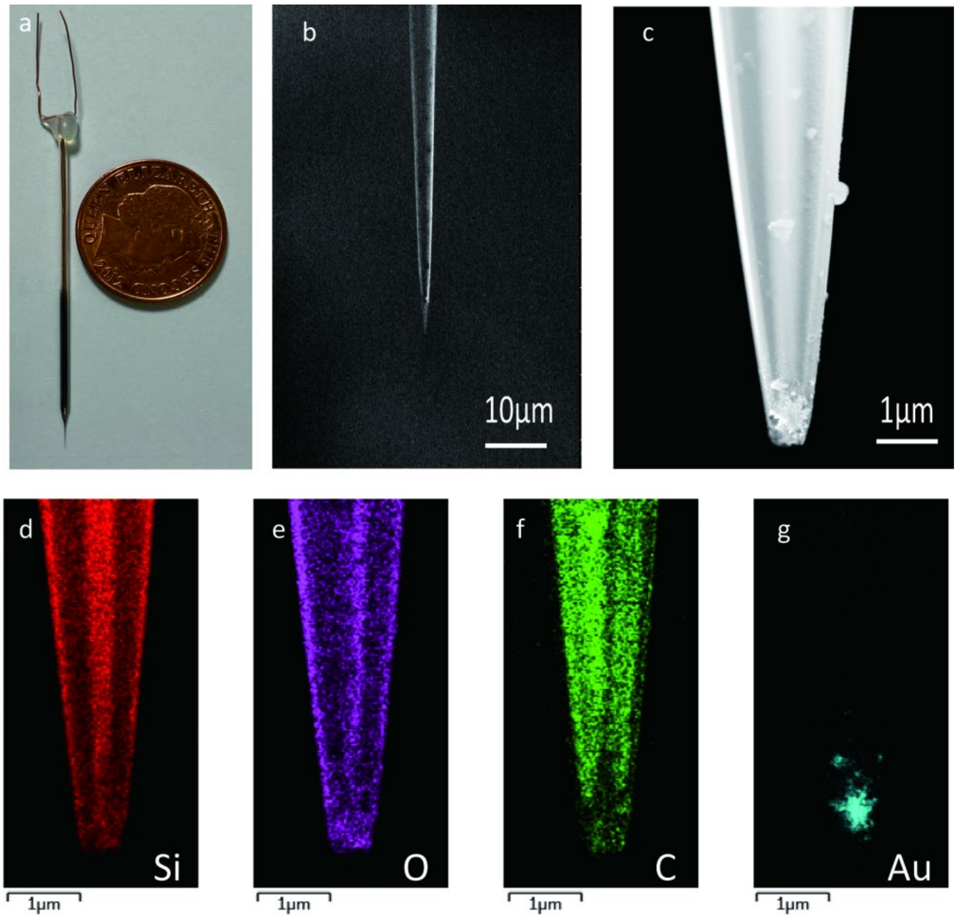

Supplementary Figure S1 microscopy characterization of a bare QMT probe. (A) Optical image (B) SEM image obtained using Zeiss Gemini Sigma 300 field emission scanning electron microscope at an acceleration voltage of 5 kV (C-G) STEM-EDX mapping. The STEM-EDX mapping shows the presence of silicon, deposited carbon, and electrodeposited gold at the QMT probe tip. Scale bar, 50 nm. The tip of the QMT probes was sliced by a focused ion beam (Quanta 3D FEG). STEM-EDX mapping was performed using a Tecnai G2 F20 TEM (FEI), equipped with Energy-dispersive X-ray spectroscopy (EDX).

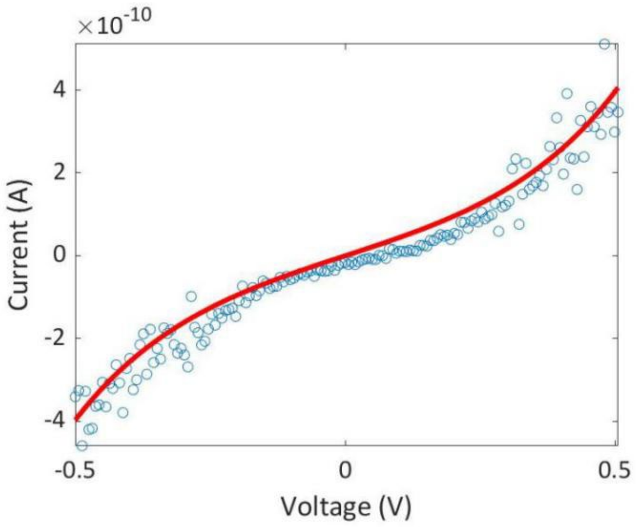

Supplementary Figure S2 The I-V curves for the different QMT devices were recorded in air and fit using the Simmons model (Equation 1).[13]

$$I = A\left(\frac{\mathrm{e}}{4\pi^2\hbar s^2}\right)\left\{\bar{\Phi}\exp\left[-\frac{2(2\mathrm{m_e})^{\frac{1}{2}}}{\hbar}\sqrt{\bar{\phi}s}\right] - (\bar{\phi}+\mathrm{eV})\exp\left[-\frac{2(2\mathrm{m_e})^{\frac{1}{2}}}{\hbar}\sqrt{\bar{\phi}+\mathrm{eVs}}\right]\right\} + B$$

Equation (1)

with bias V, work function ϕ, barrier height Φ_B, gap distance d, and $\bar{\Phi} = \Phi_\mathrm{B}/2$ and $s = d\Phi_\mathrm{B}/\mathrm{eV}$, A the active tunneling area, and B is a parameter that considers any current offset at zero bias due to residual Faradaic processes or minor calibration errors

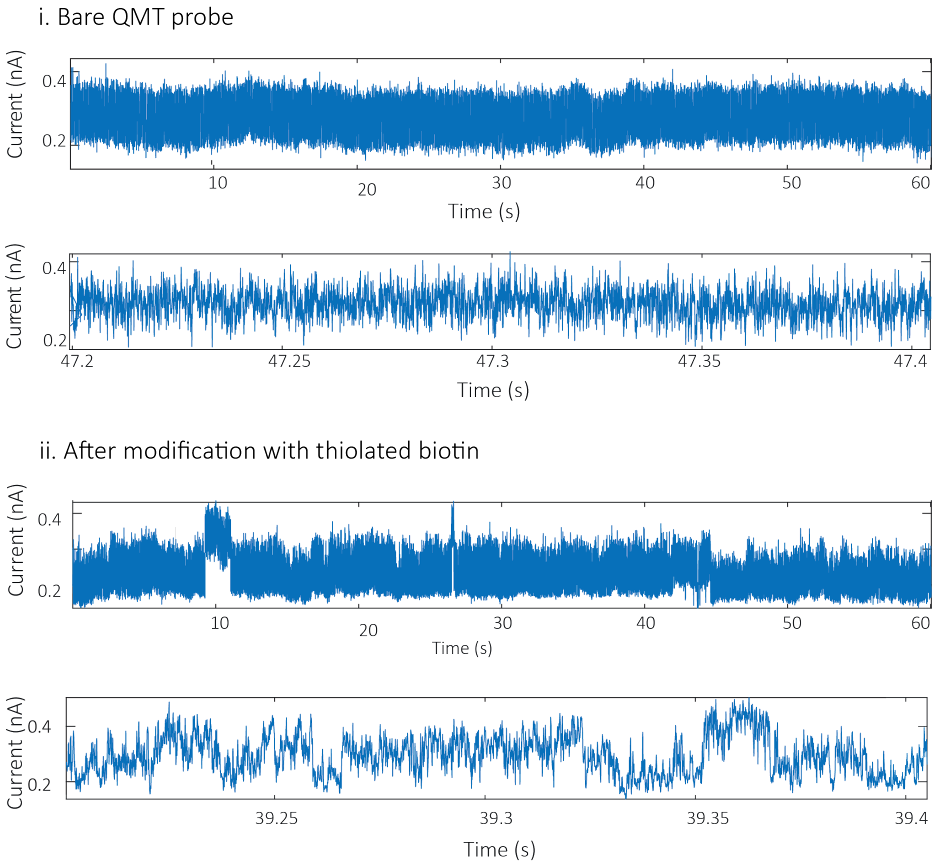

Supplementary Figure S3 The real-time chronoamperometric recording of the current for a bare QMT probe before (i) and after (ii) modification with thiolated biotin in 1 mM PBS (pH 7.4) at 100 mV.

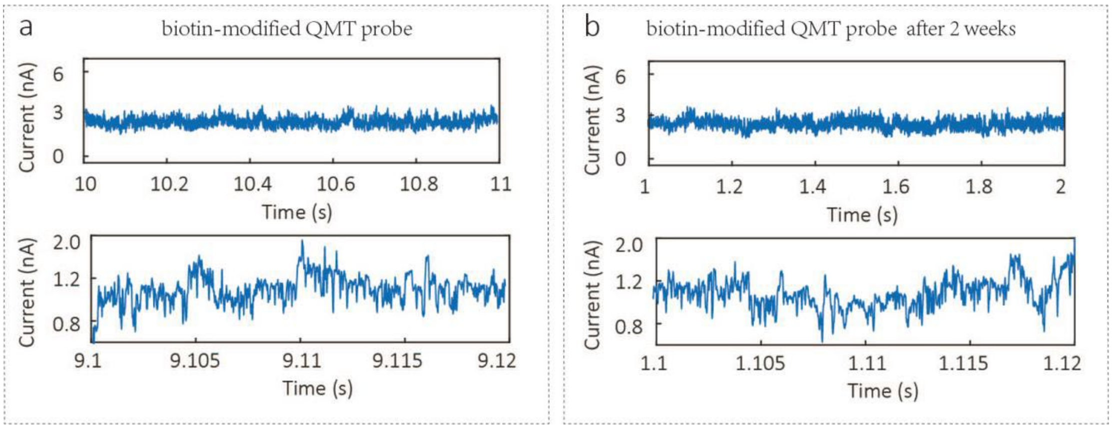

Supplementary Figure S4 Long-term stability of the biotin-modified QMT probes. Current-time Measurements were performed in 1 mM PBS at 100 mV over 1-2 week period.

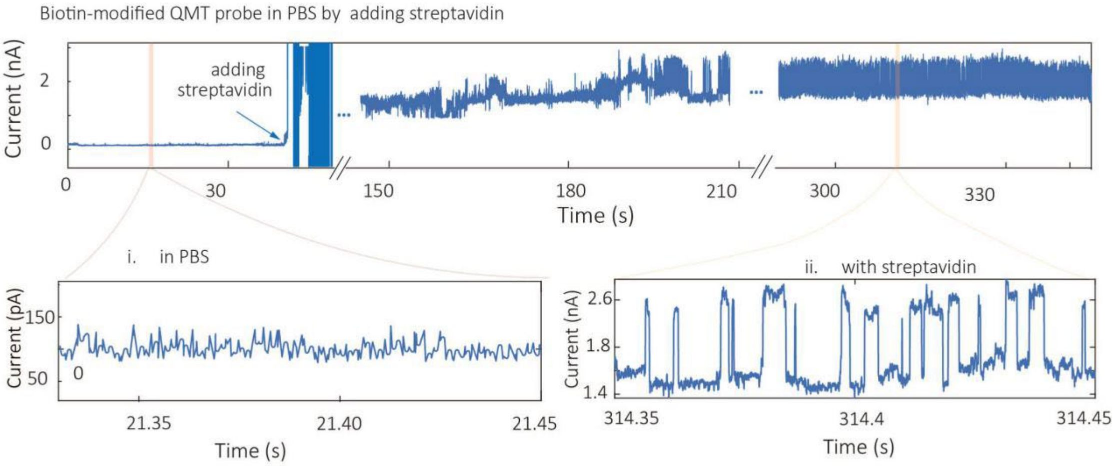

Supplementary Figure S5 Real-time tunneling current of biotin-QMT probe recorded at 100 mV in 1 mM PBS (pH 7.4) after the addition of 0.2 ng/mL streptavidin. Frequent transient events with two well-defined levels of increased tunneling current were observed.

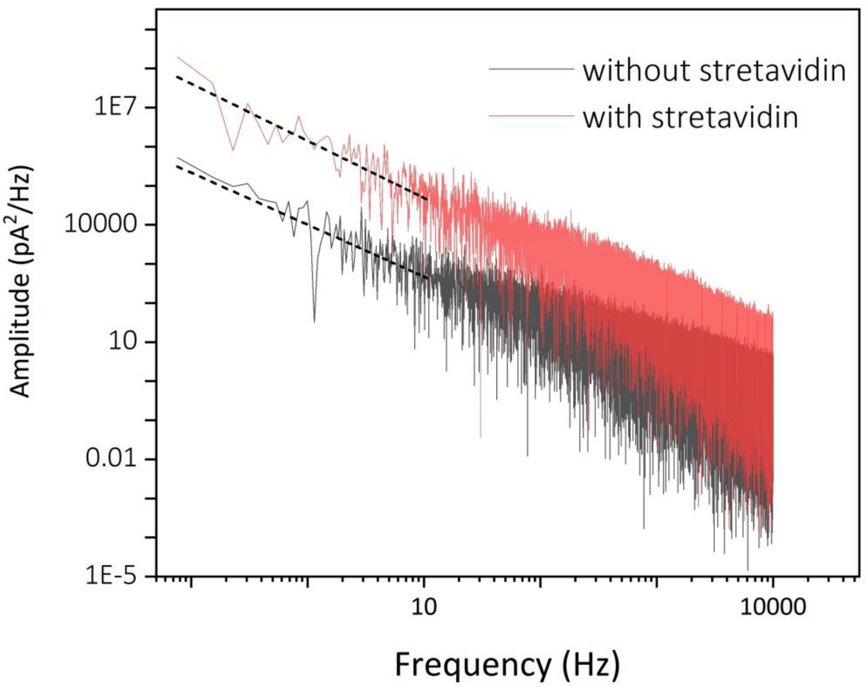

Supplementary Figure S6 Current noise analysis of biotin-modified QMT probes before (black) and after (red) adding streptavidin. The current-time signals correspond to the biotin-modified QMT probes without (Figure 2E ii) and with streptavidin (Figure 2E iii), which were recorded for 1 s with a patch-clamp amplifier at 100 mV bias. The dashed lines were fitted to $S^2(f) \propto 1/f$.

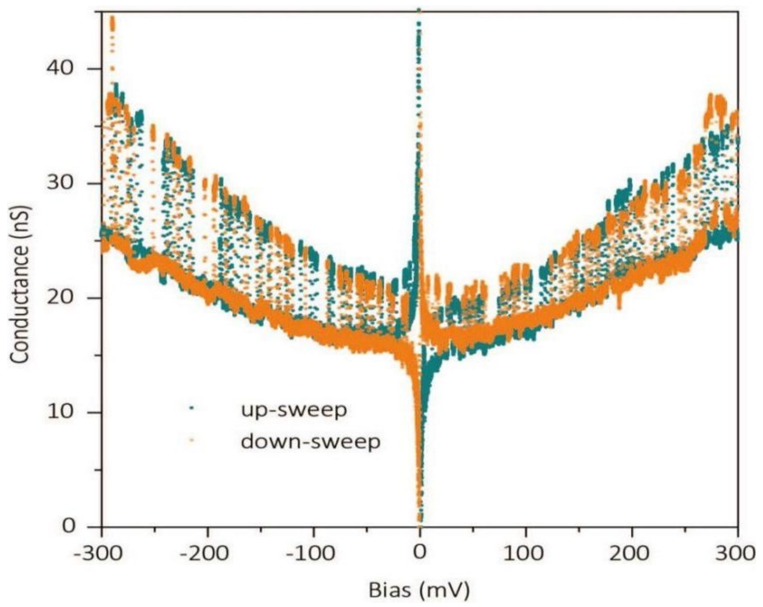

Supplementary Figure S7 Conductance-bias plot of streptavidin-coupled QMT probe corresponding to Figure 2G.

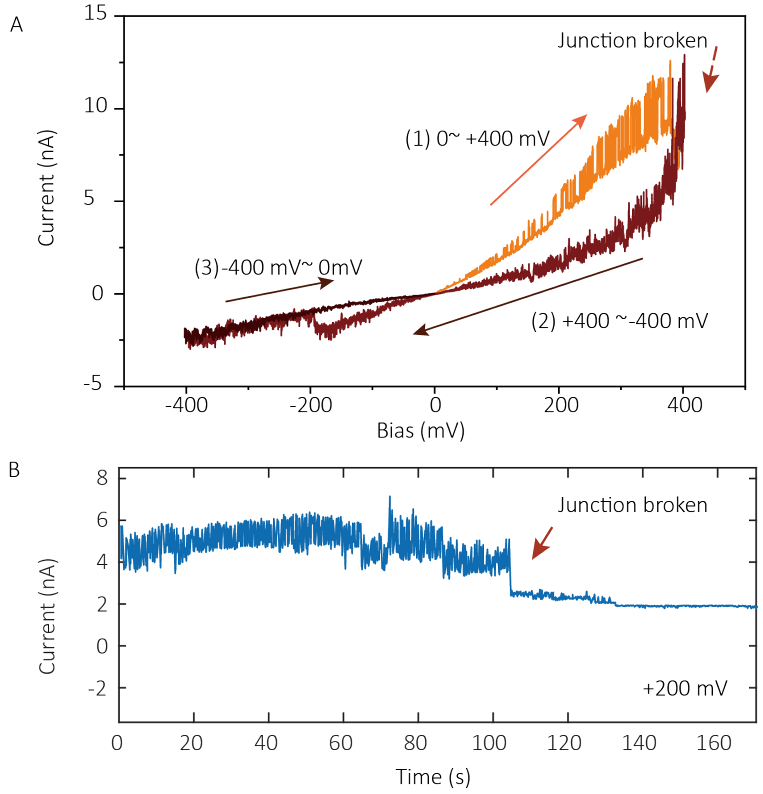

Supplementary Figure S8 (A) Current-bias profile recorded for streptavidin-coupled QMT probe in the 1 mM PBS. (B) Current-time trace of streptavidin-coupled QMT probe in the 1 mM PBS measured at +200 mV. A sudden decrease in the current indicates the potential breaking of the biotin-SA-biotin junction.

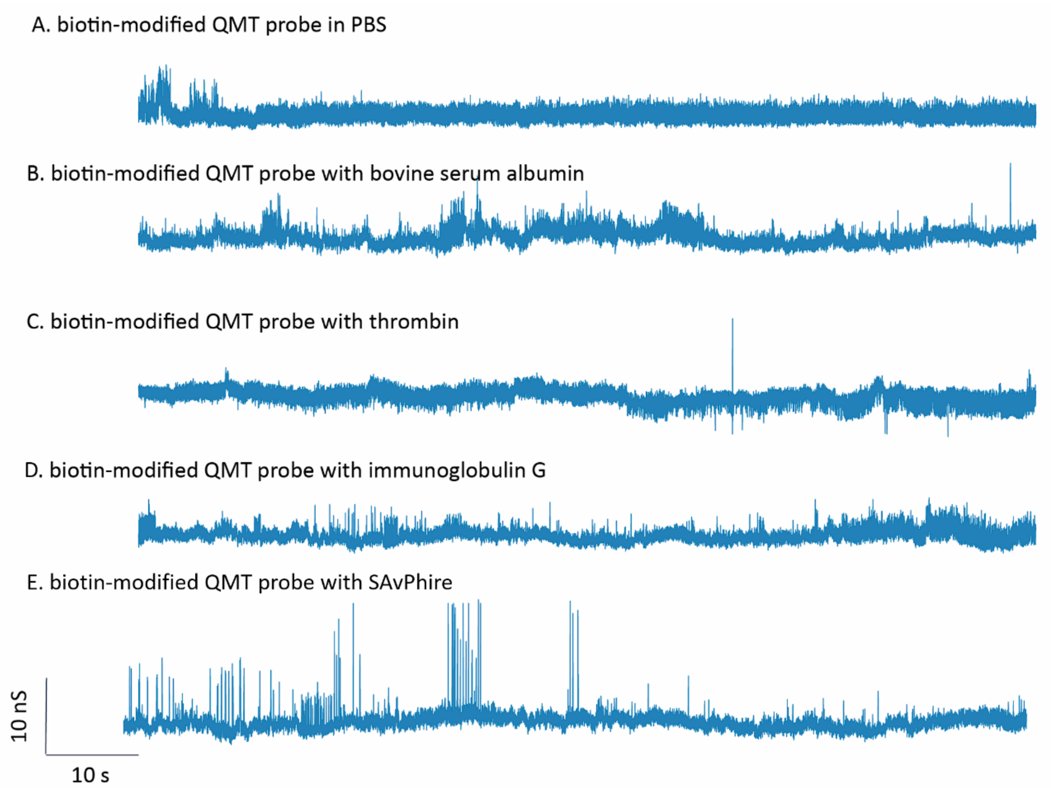

Supplementary Figure S9 Current recording for biotin-modified QMT probes in 1 mM PBS (A) before and (B-E) after adding different proteins to confirm selective binding. When 0.2 ng/mL immunoglobulin G bovine serum albumin and thrombin were added, no significant current transients were observed, with only very few spike-like signals; while in the presence of the monomeric SA isotype with only one binding site to biotin (SAvPhire from Sigma-Aldrich), the current rise could be observed on biotin-modified QMT probes. Bias: 100 mV.

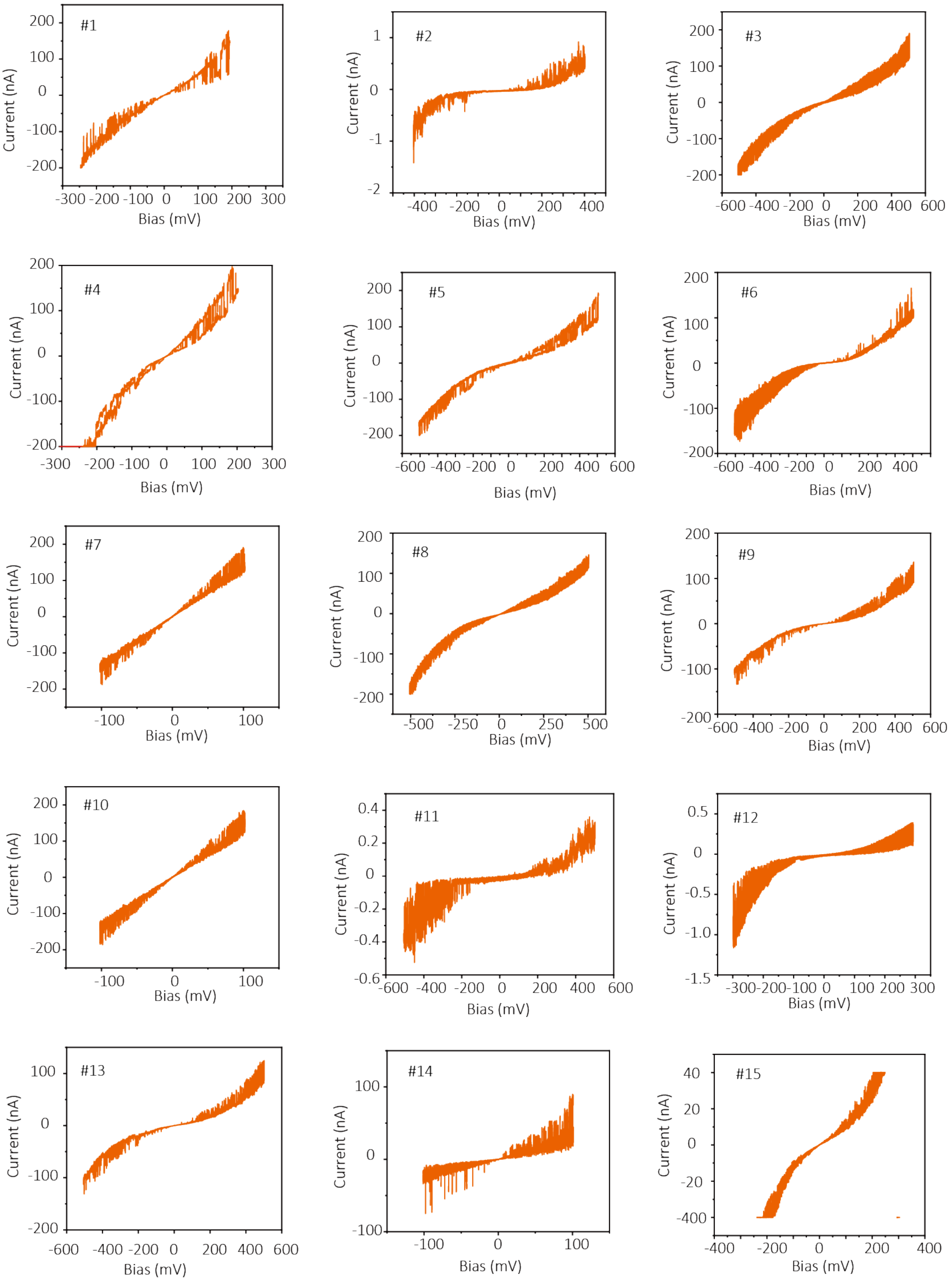

Supplementary Figure S10 Current-bias plots of different streptavidin-coupled QMT probes measured in 1 mM phosphate buffer solution (PBS) at room temperature (297 K).

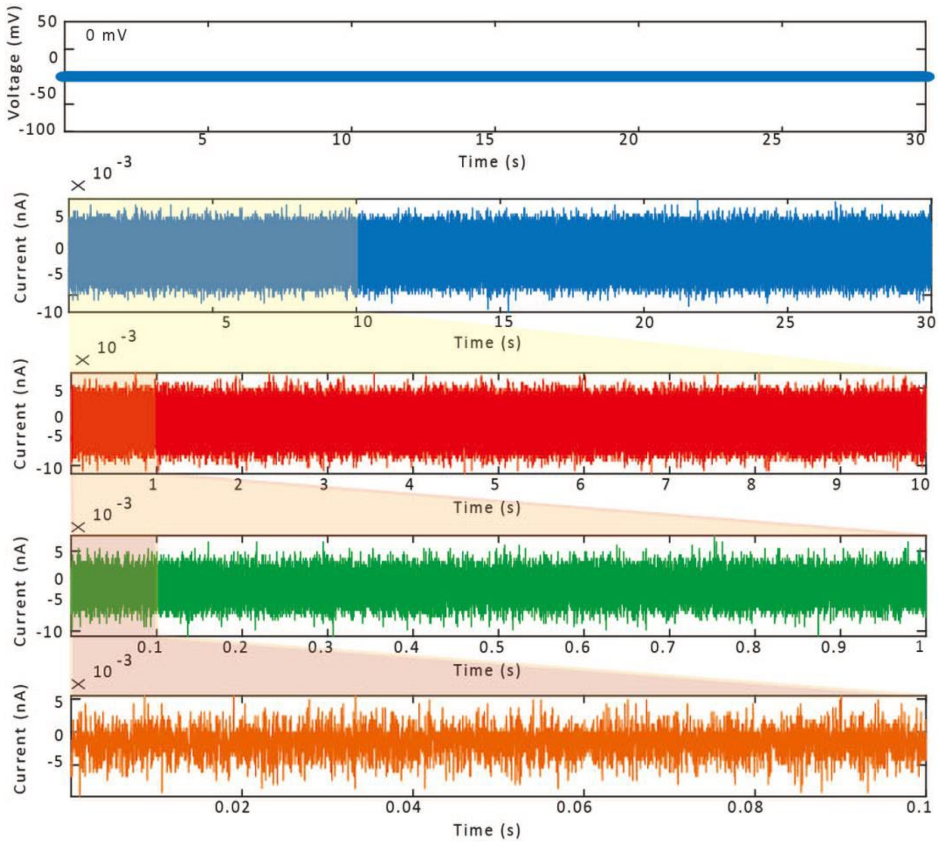

A. Bias at 0 mV

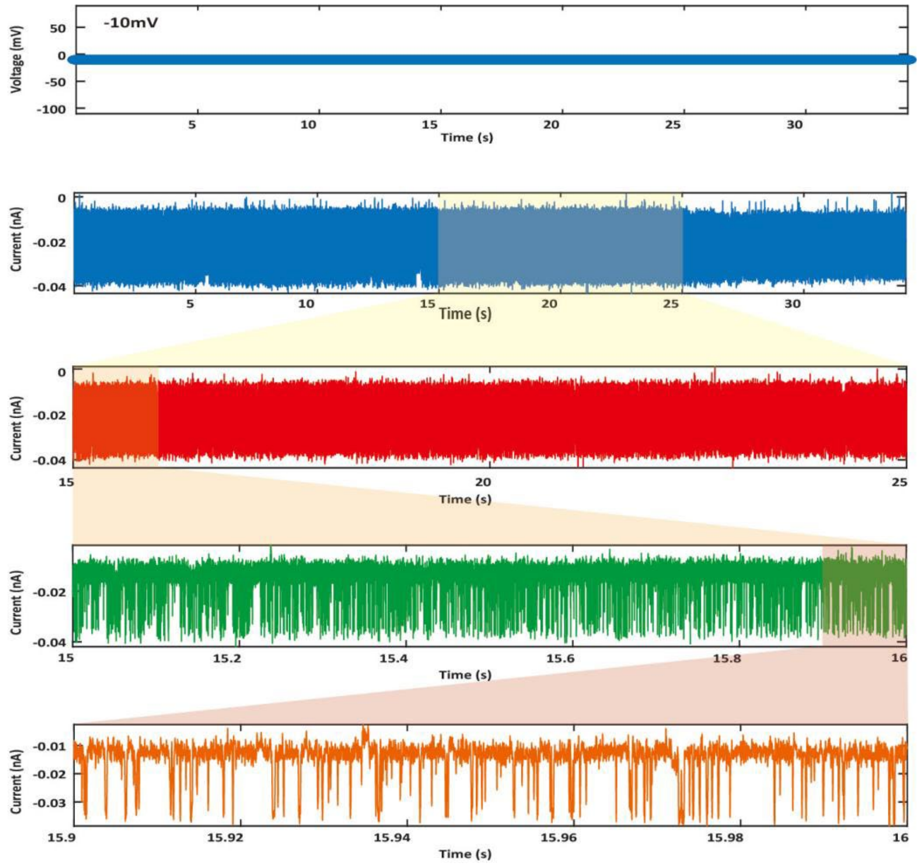

B. Bias at -10 mV

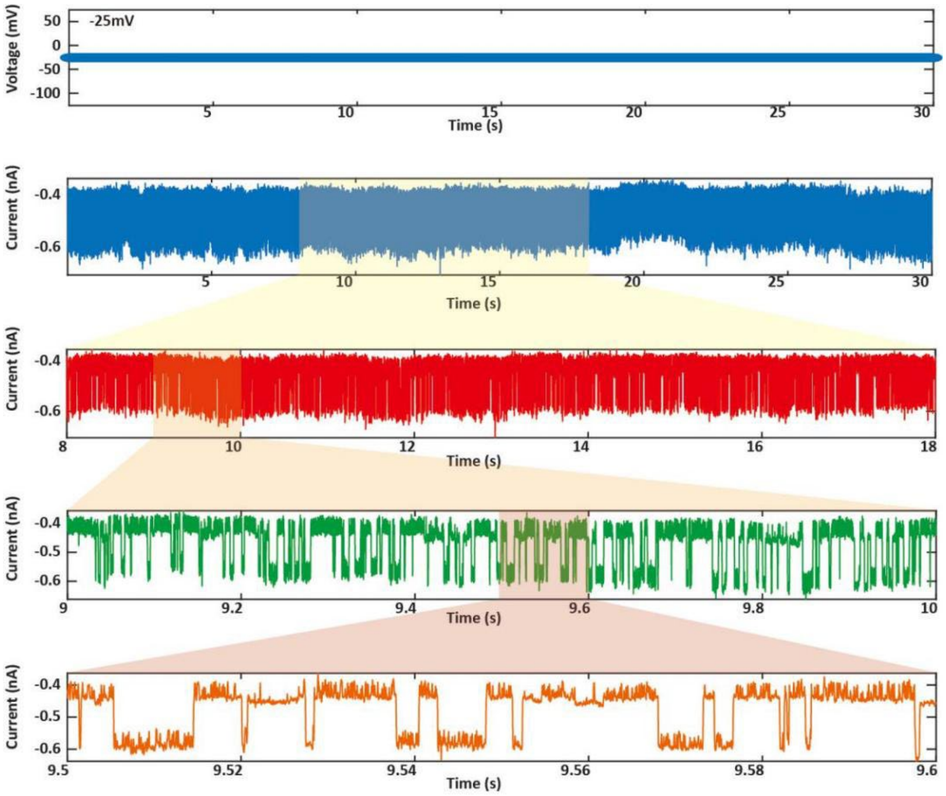

C. Bias at -25 mV

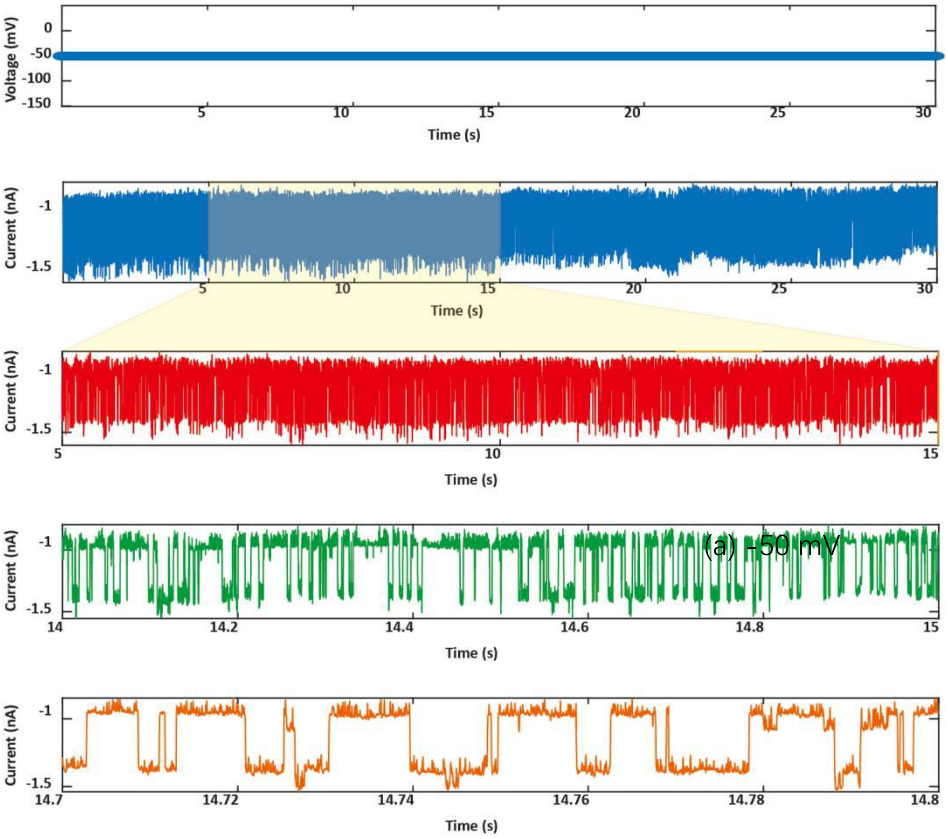

D. Bias at -50 mV

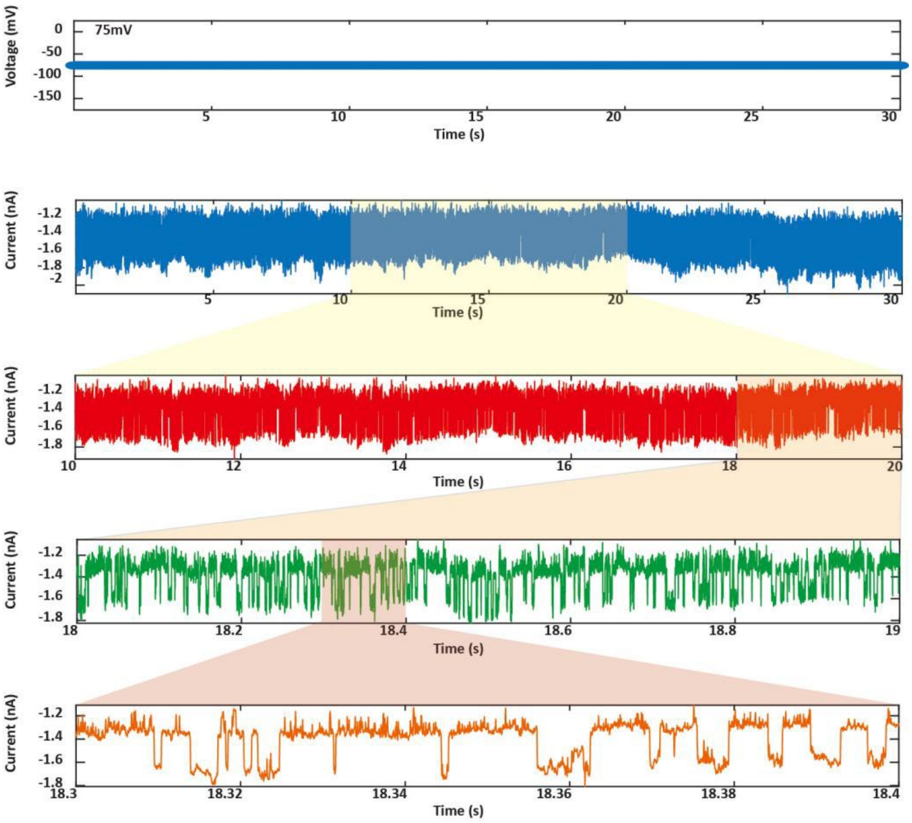

E. Bias at -75 mV

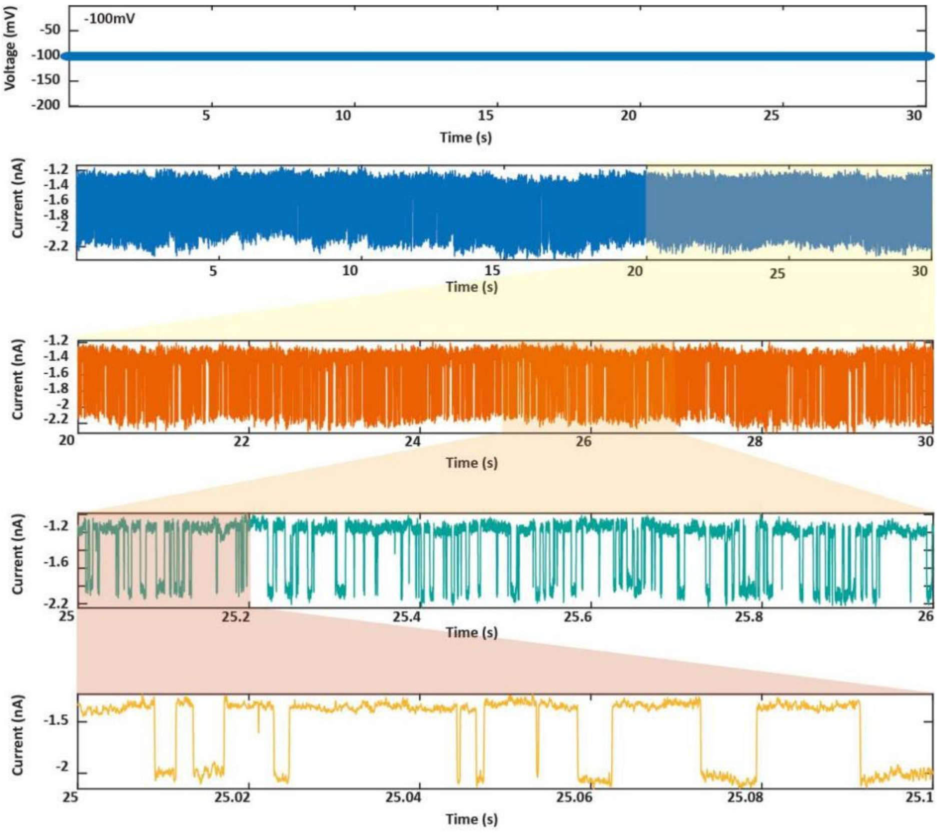

F. Bias at -100 mV

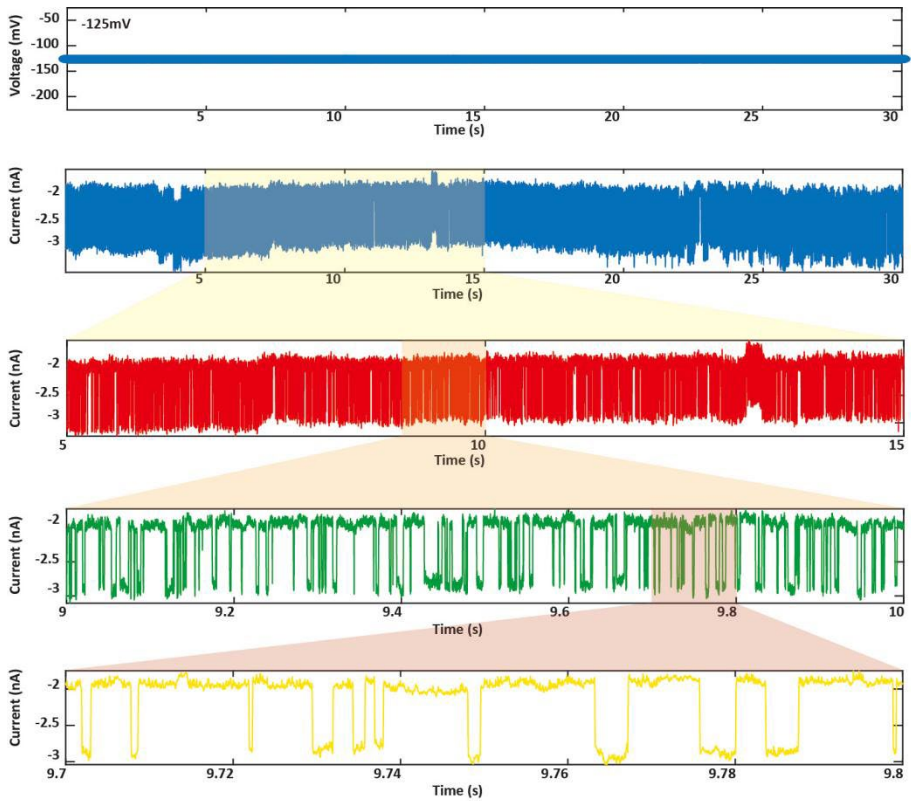

G. Bias at -125 mV

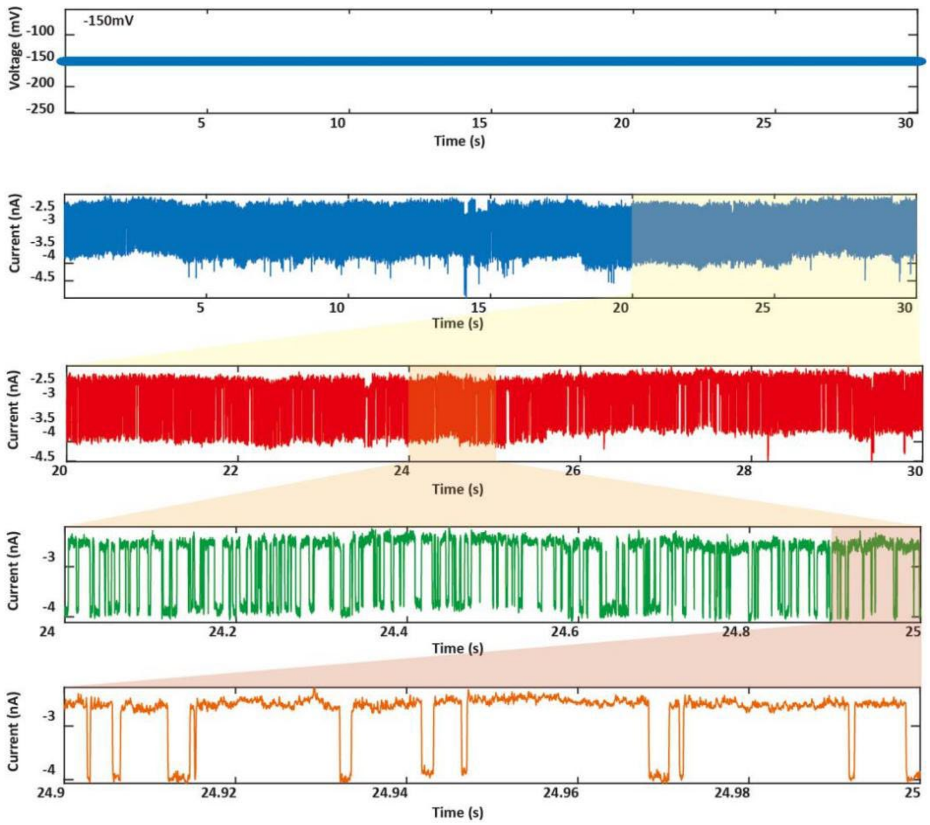

H. Bias at -150 mV

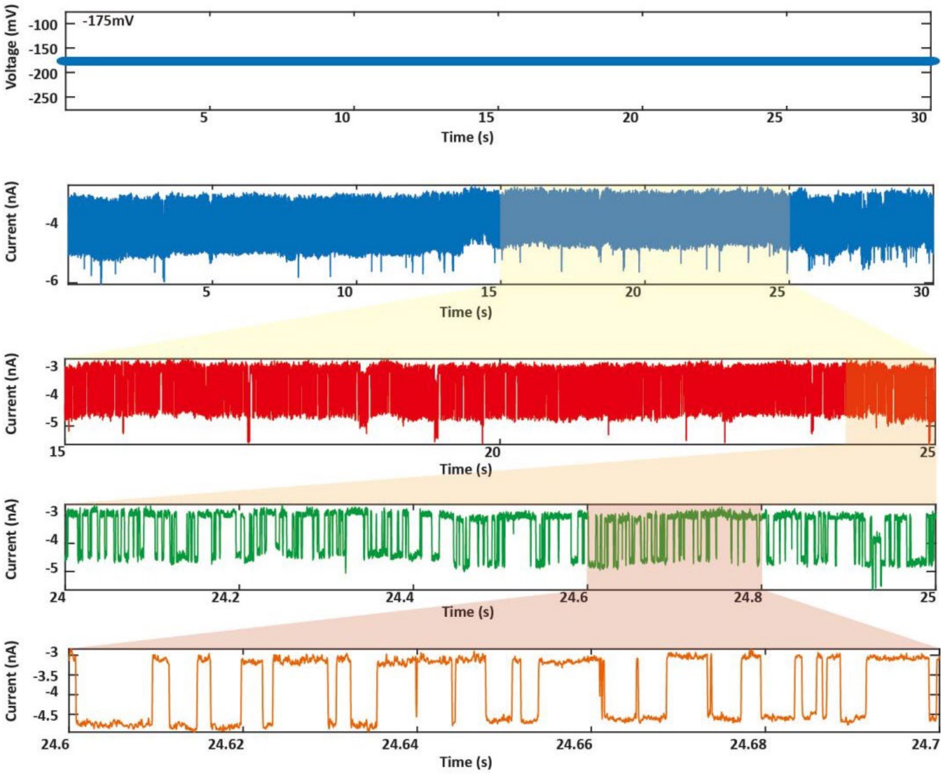

I. Bias at -175 mV

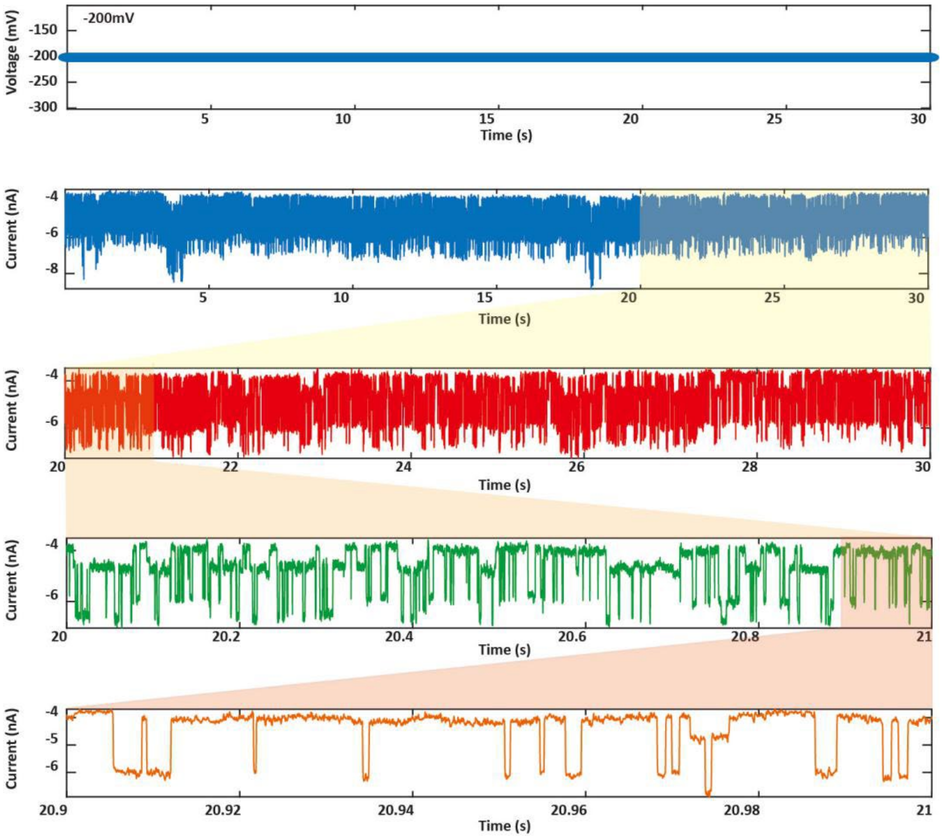

J. Bias at -200 mV

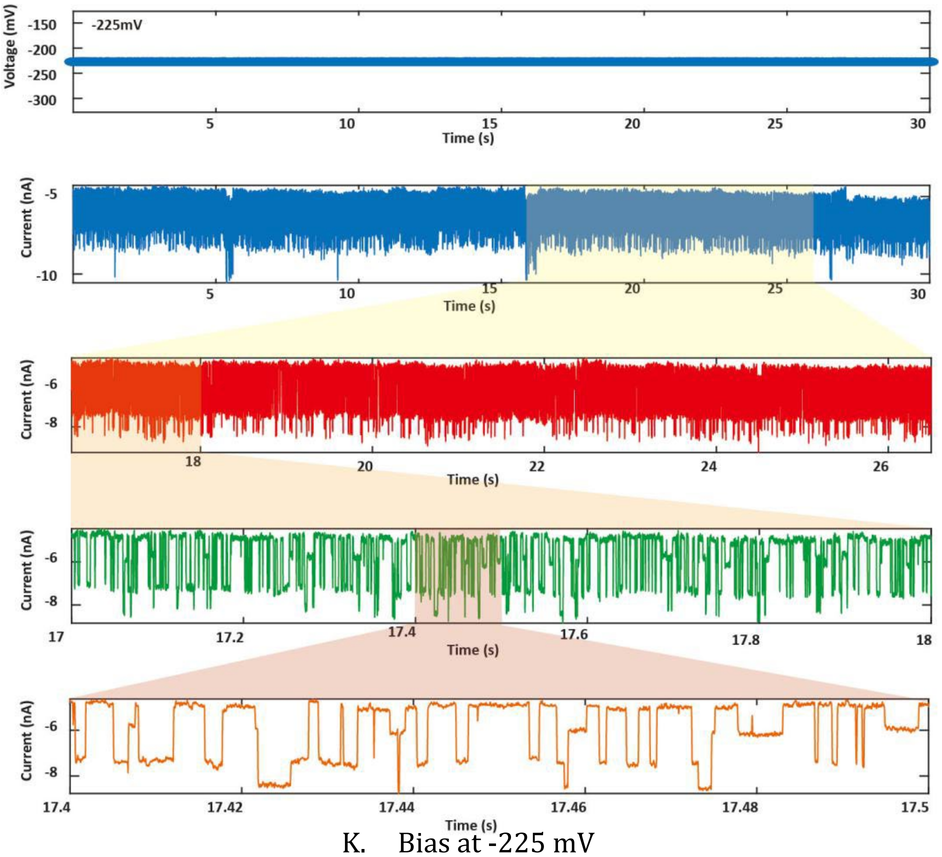

K. Bias at -225 mV

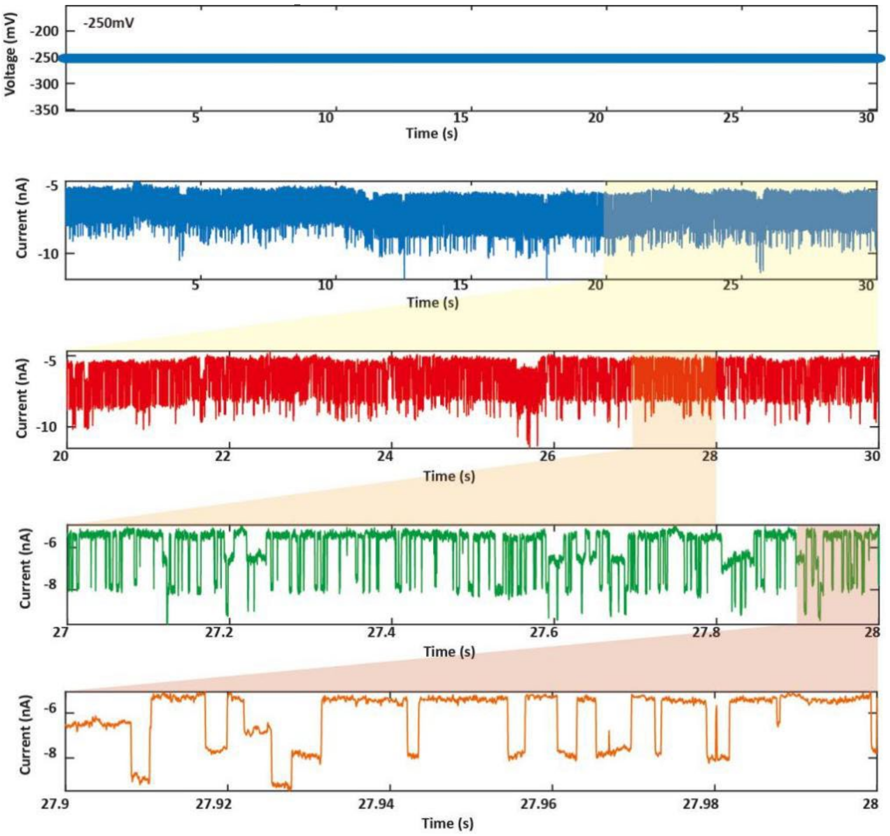

M. Bias at-250 mV

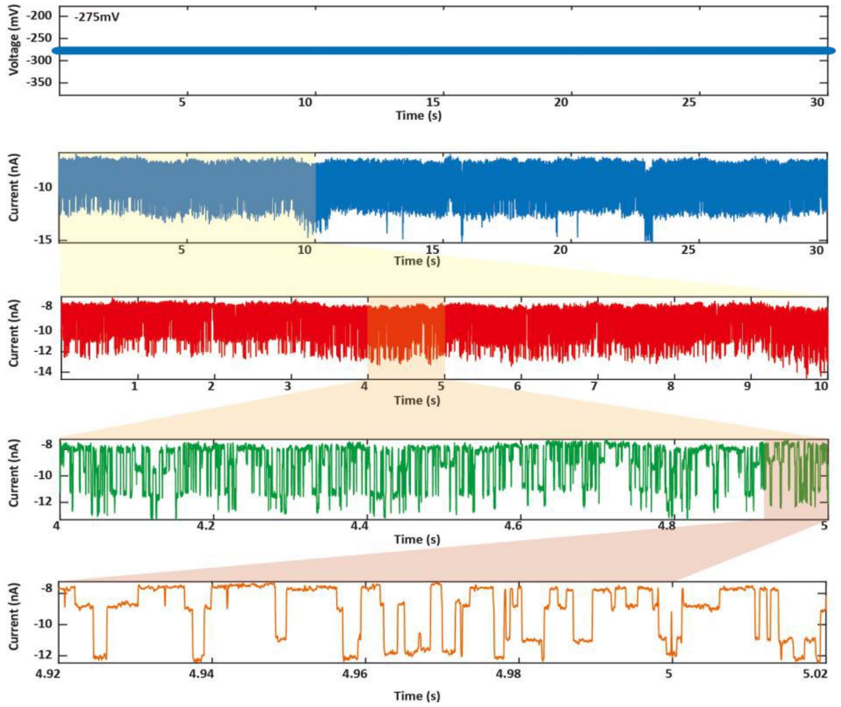

N. Bias at -275 mV

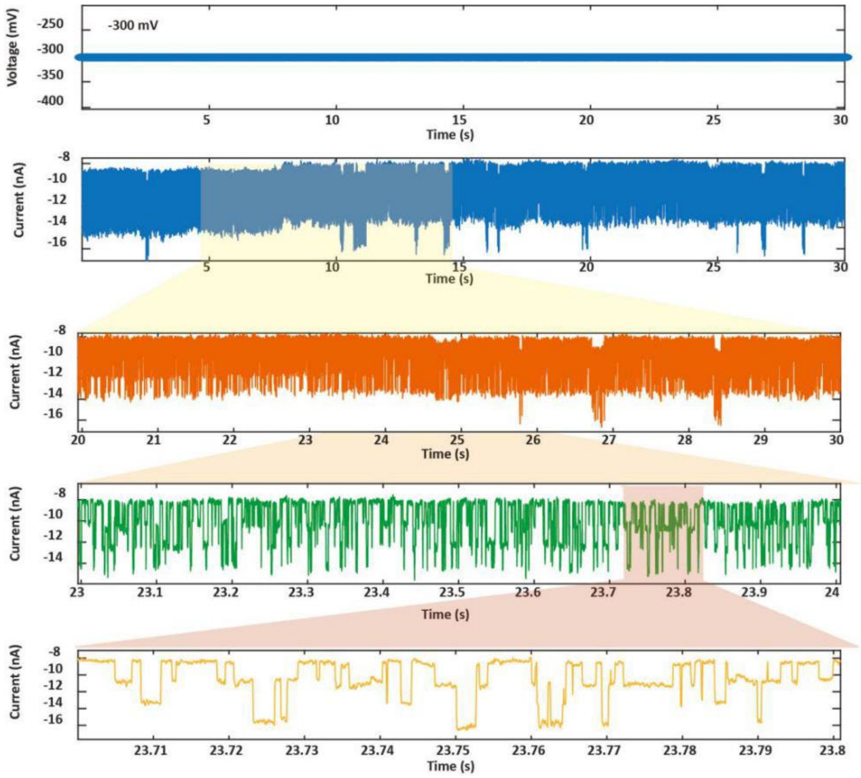

O. Bias at -300 mV

Supplementary Figure S11 (A-N) Chronoamperometric traces of streptavidin-coupled QMT probes between 0 and -300 mV in1 mM PBS at room temperature.

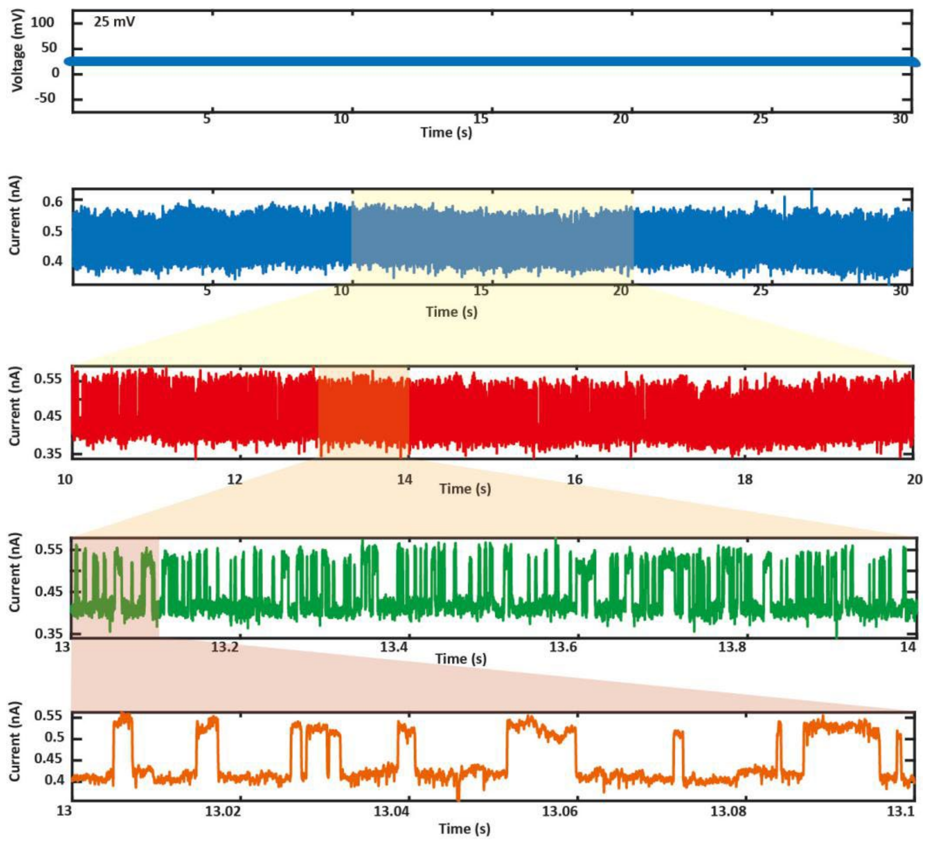

A. Bias at +25 mV

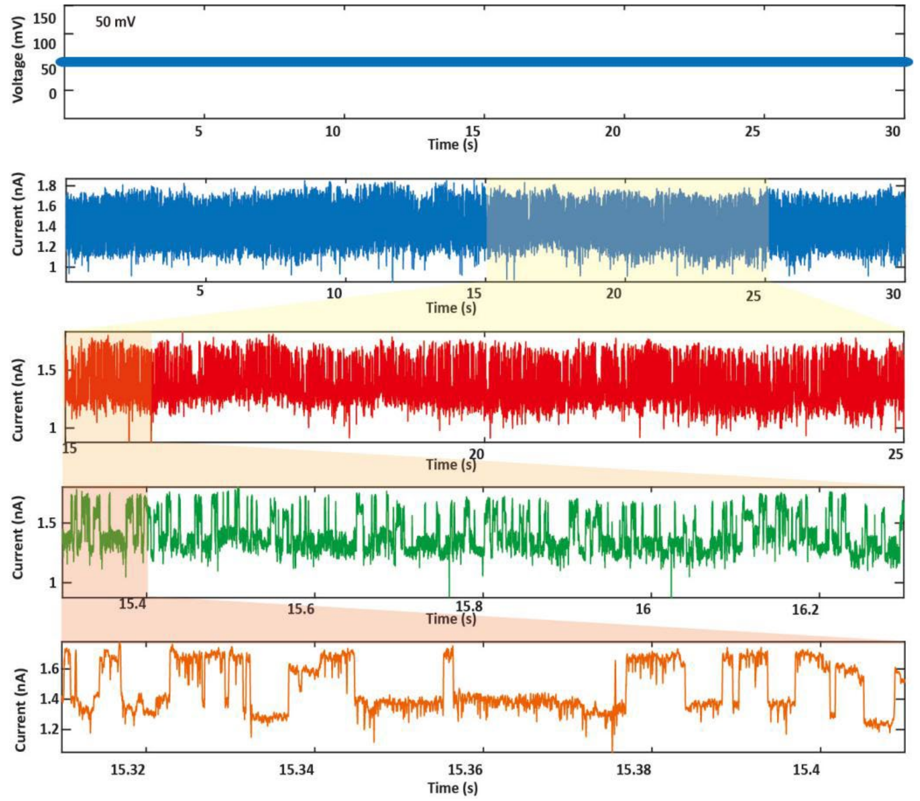

B. Bias at +50 mV

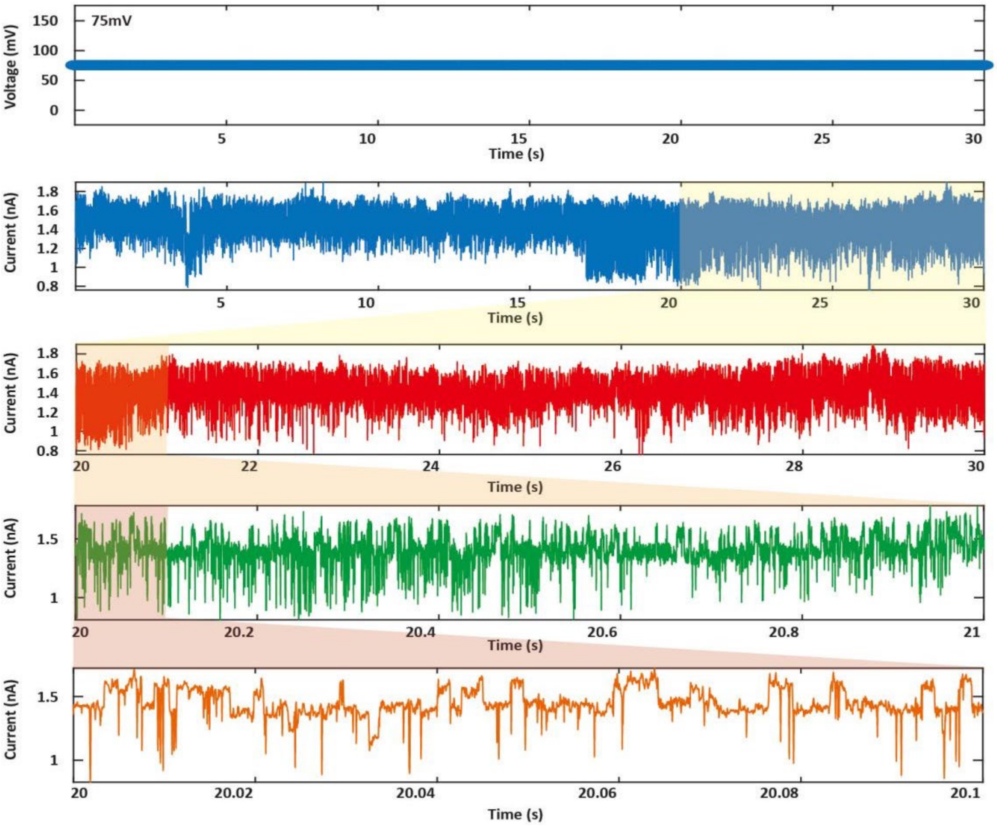

C. Bias at +75 mV

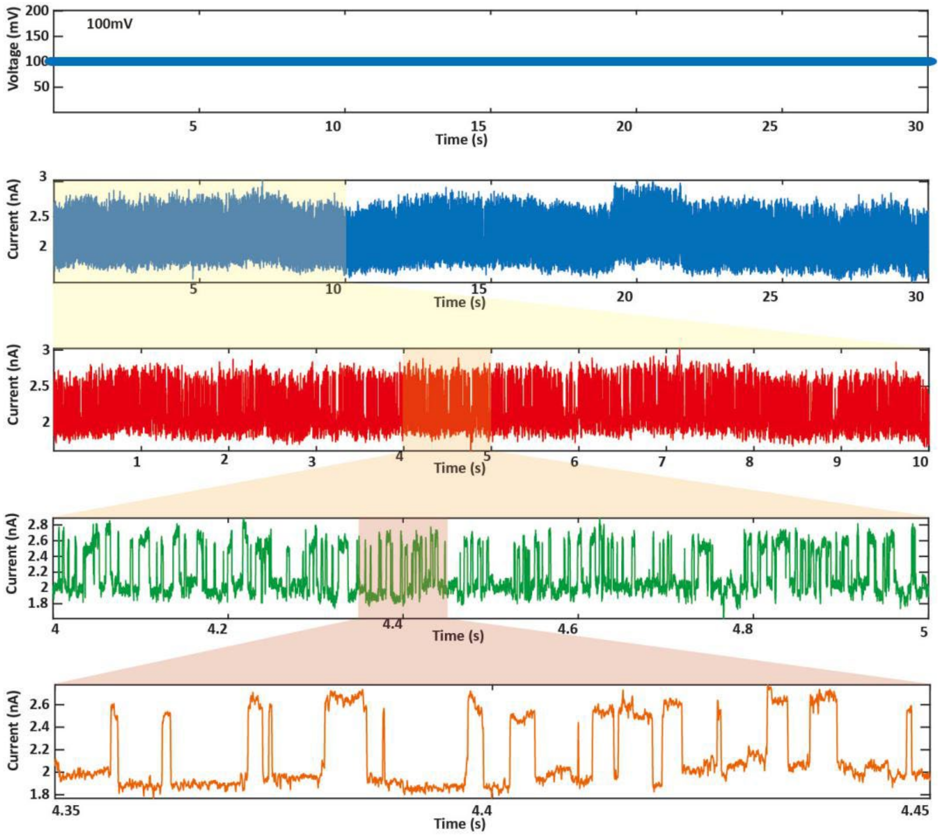

D. Bias at +100 mV

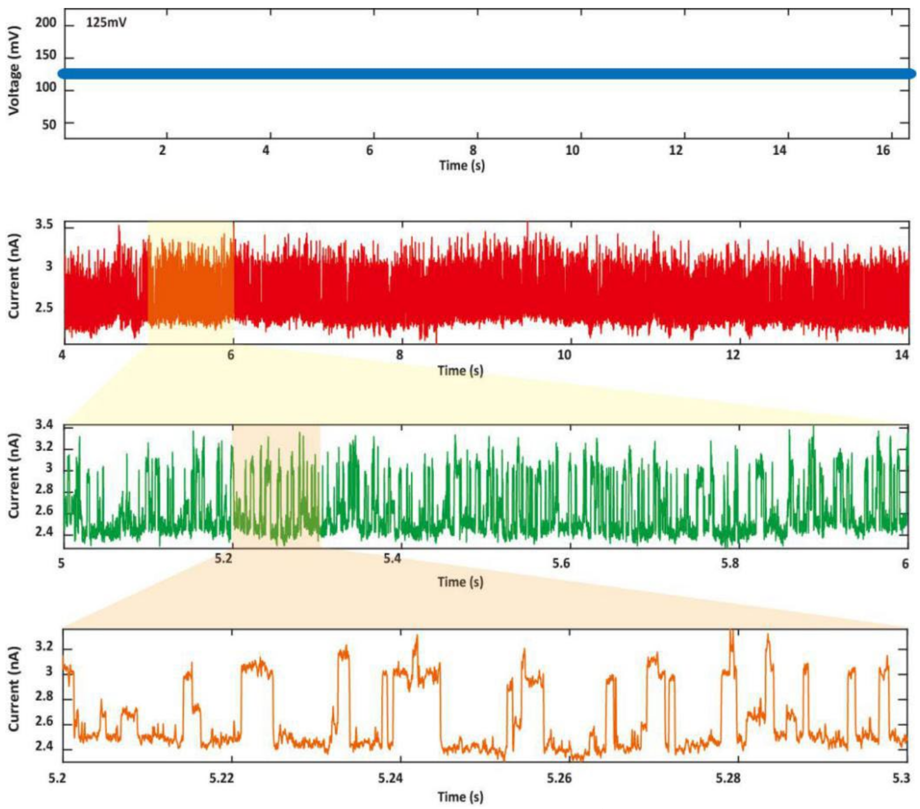

E. Bias at +125 mV

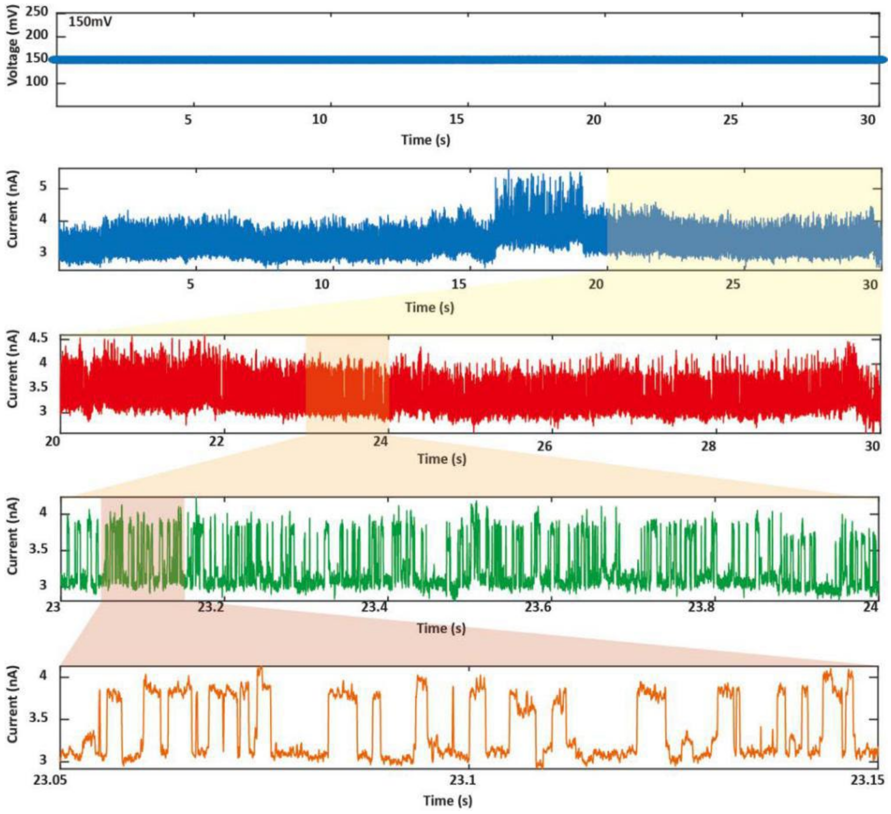

F. Bias at +150 mV

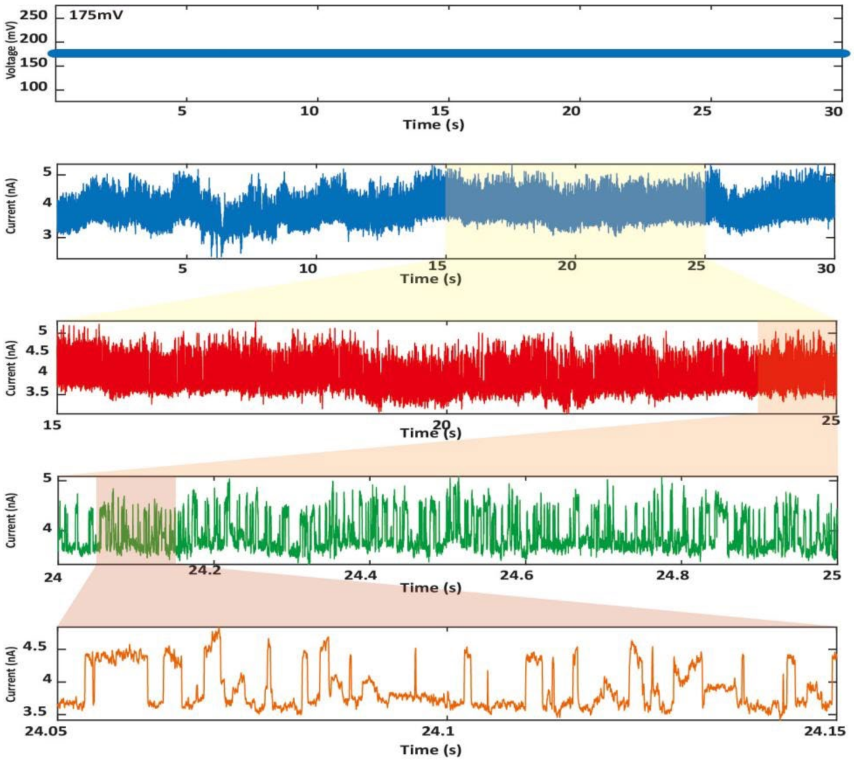

G. Bias at +175 mV

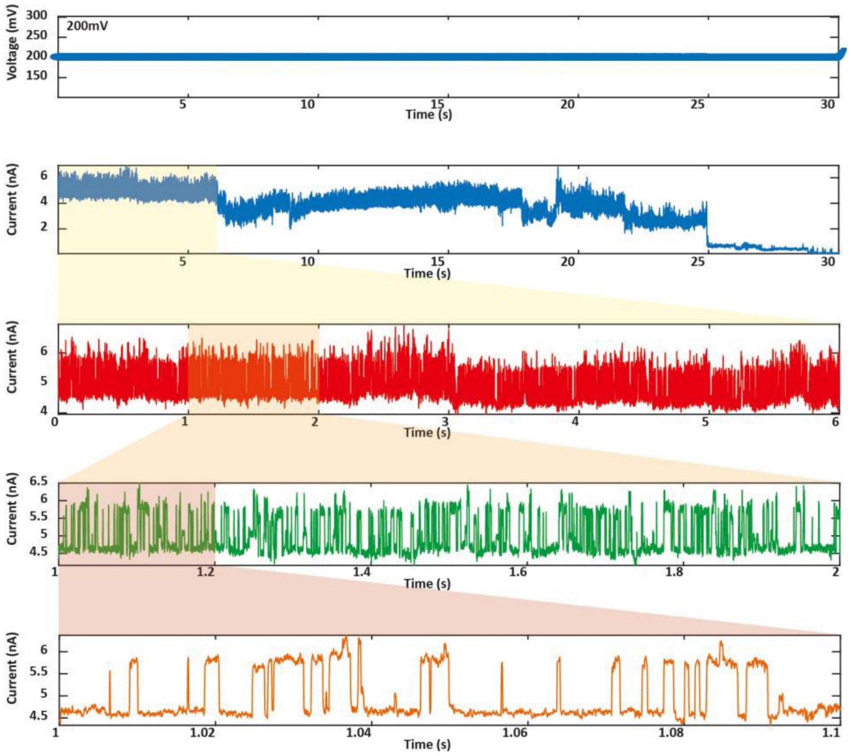

H. Bias at +200 mV

Supplementary Figure S12 (A-H) Chronoamperometric traces of streptavidin-coupled QMT probes between 25 mV and 200 mV in 1 mM PBS at room temperature.

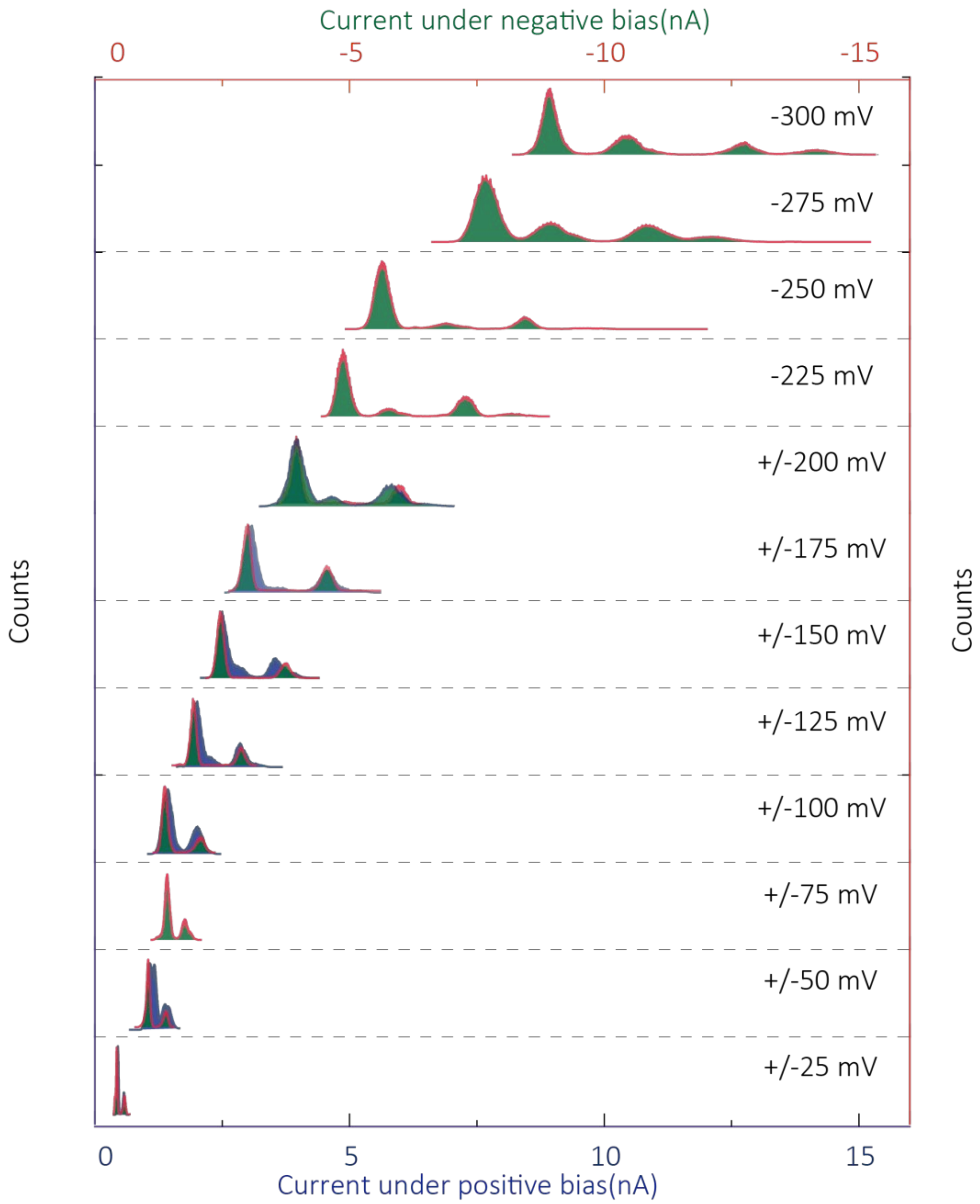

Supplementary Figure S13 Current histograms corresponding to Figure S11 and S12

.

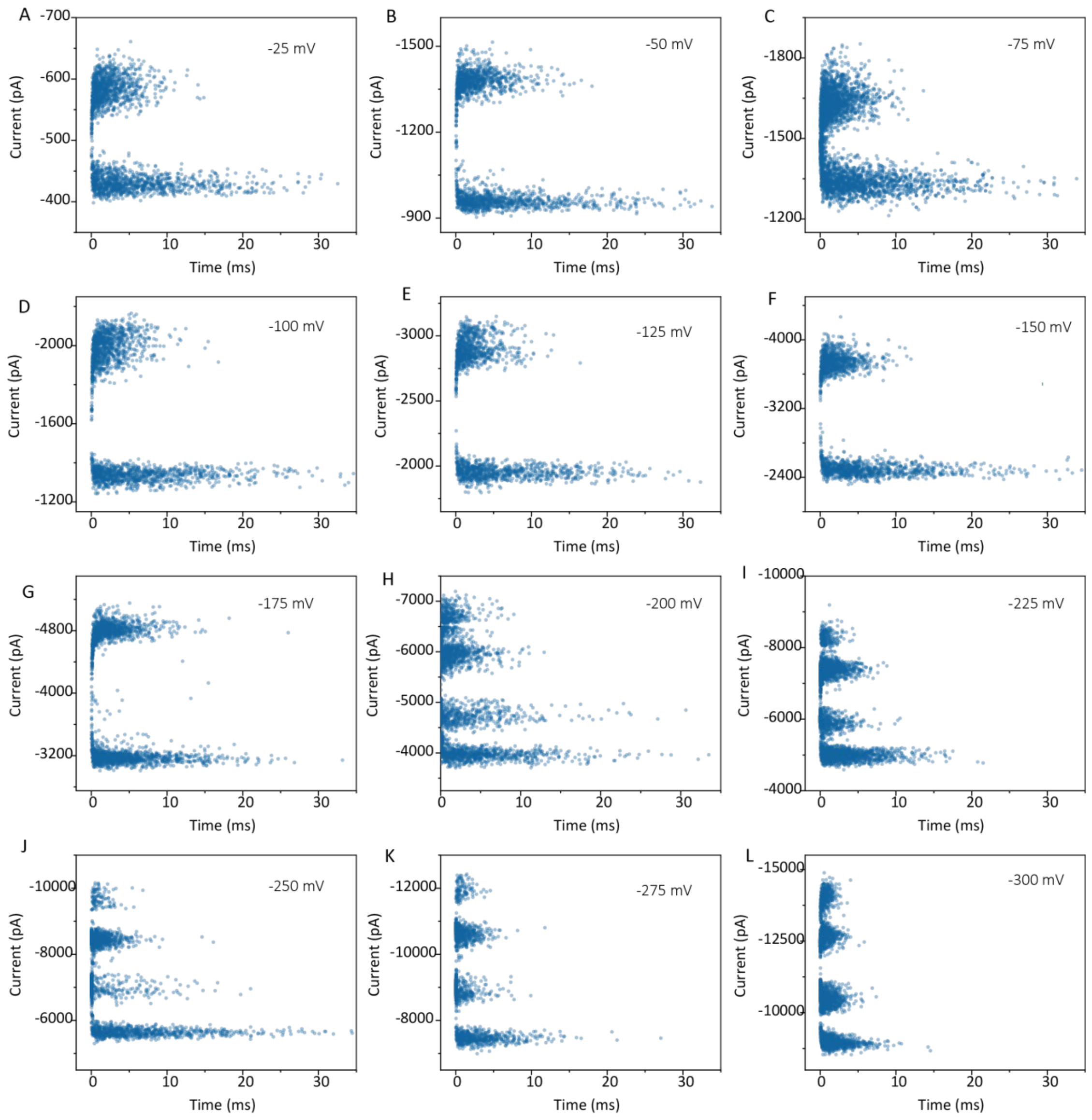

Supplementary Figure S14 Scatter plots of current amplitude and dwell time for each state at constant bias between -25 mV and -300 mV.

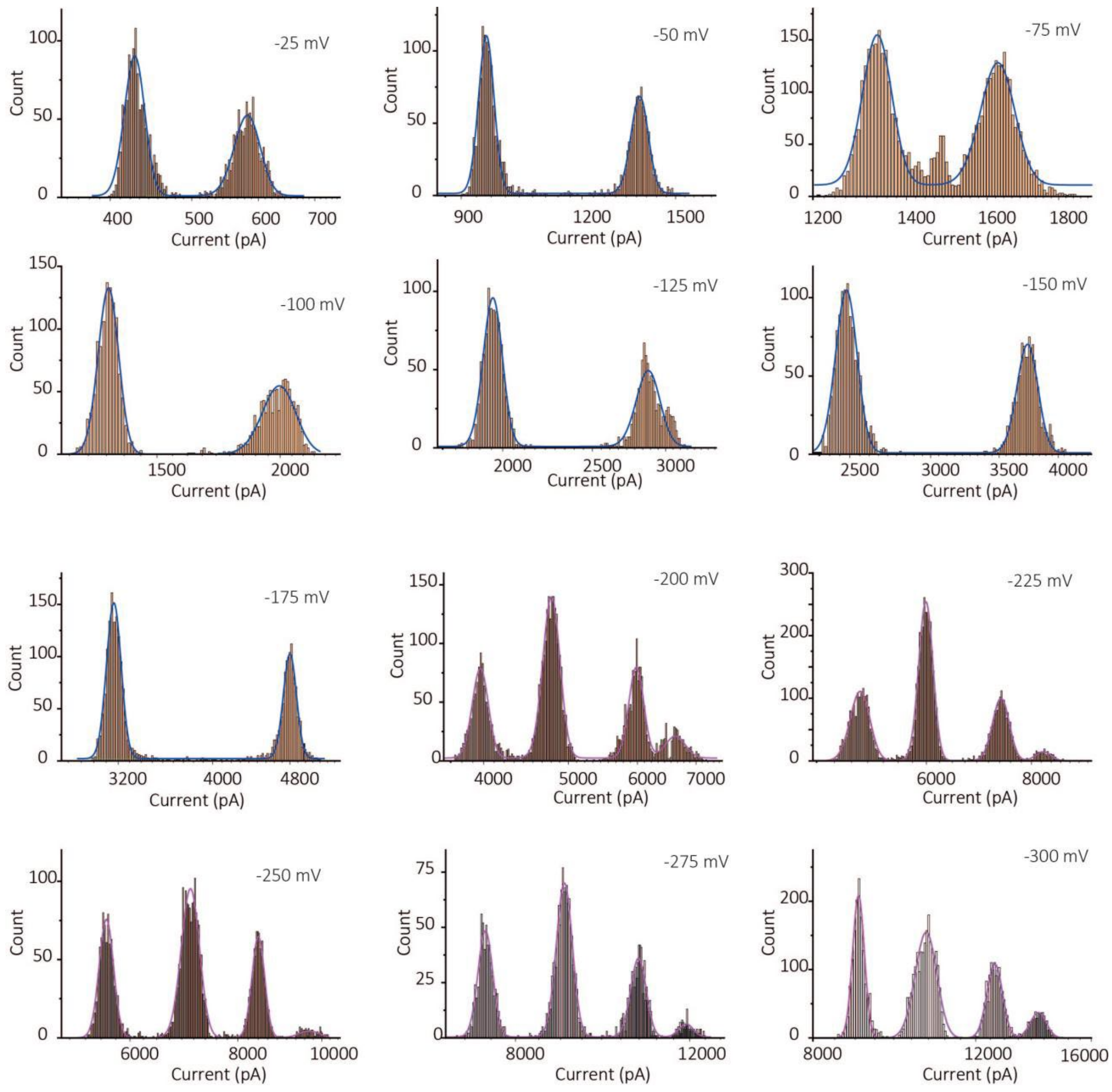

.

Supplementary Figure S15 Histogram of current amplitude for each state at constant bias between -25 and -300 mV.

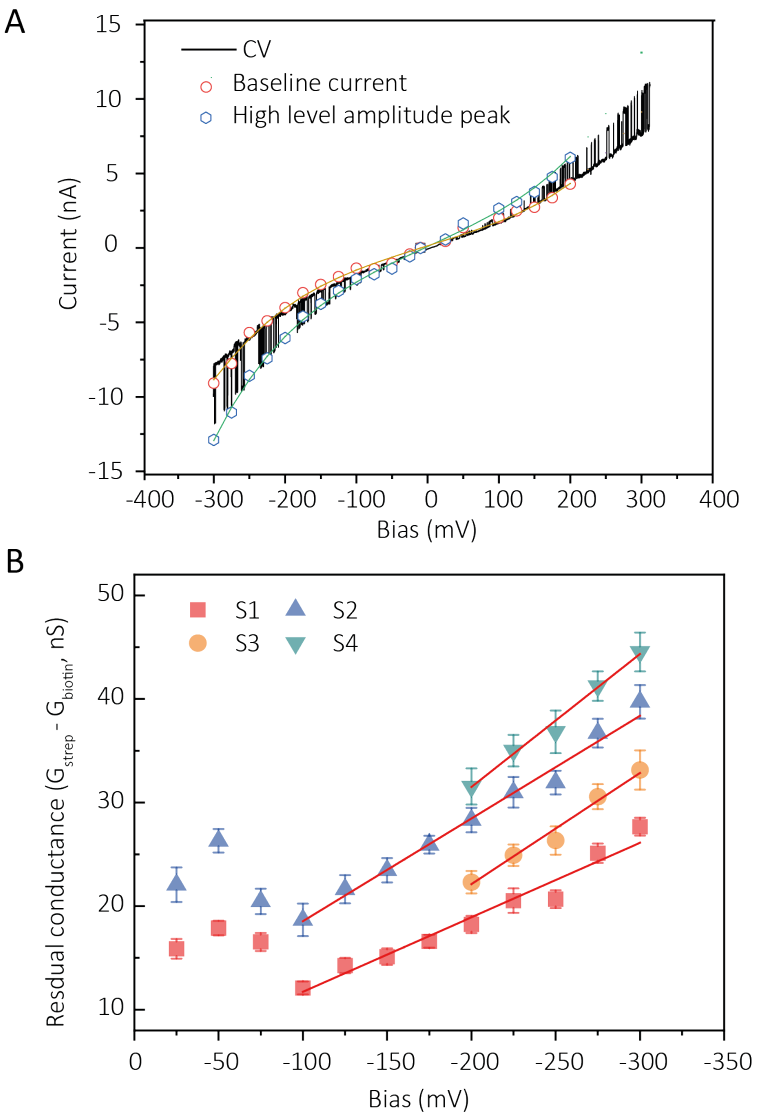

Supplementary Figure S16 (A) The conductance peaks of S1 and S2 agreed well with the I-V data in Fig. 2G. (B) Residual conductance of the streptavidin-coupled probe under different bias obtained as the difference between the conductance measured in streptavidin bound junctions (G_{strep}) and the conductance (G_{biotin}) of biotin-modified QMT probe in Fig 4D.

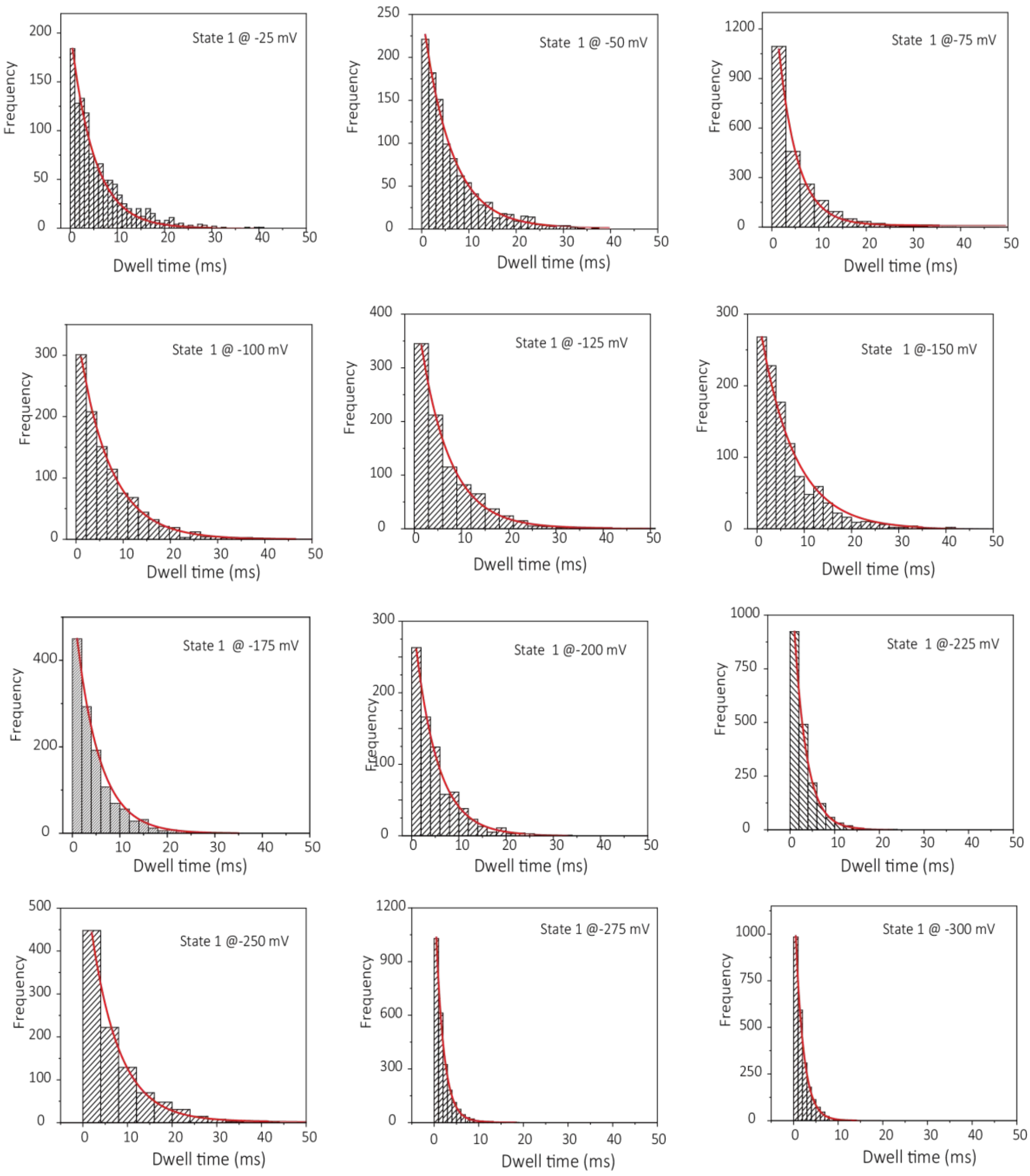

Supplementary Figure S17 Dwell time histograms for conductance state 1 for the streptavidin-coupled QMT probe in the different bias. The solid red line corresponds to monoexponential fits from which the lifetime constant τs1 has been calculated. Transition lifetimes were obtained by a monoexponential fit of the dwell time histogram.

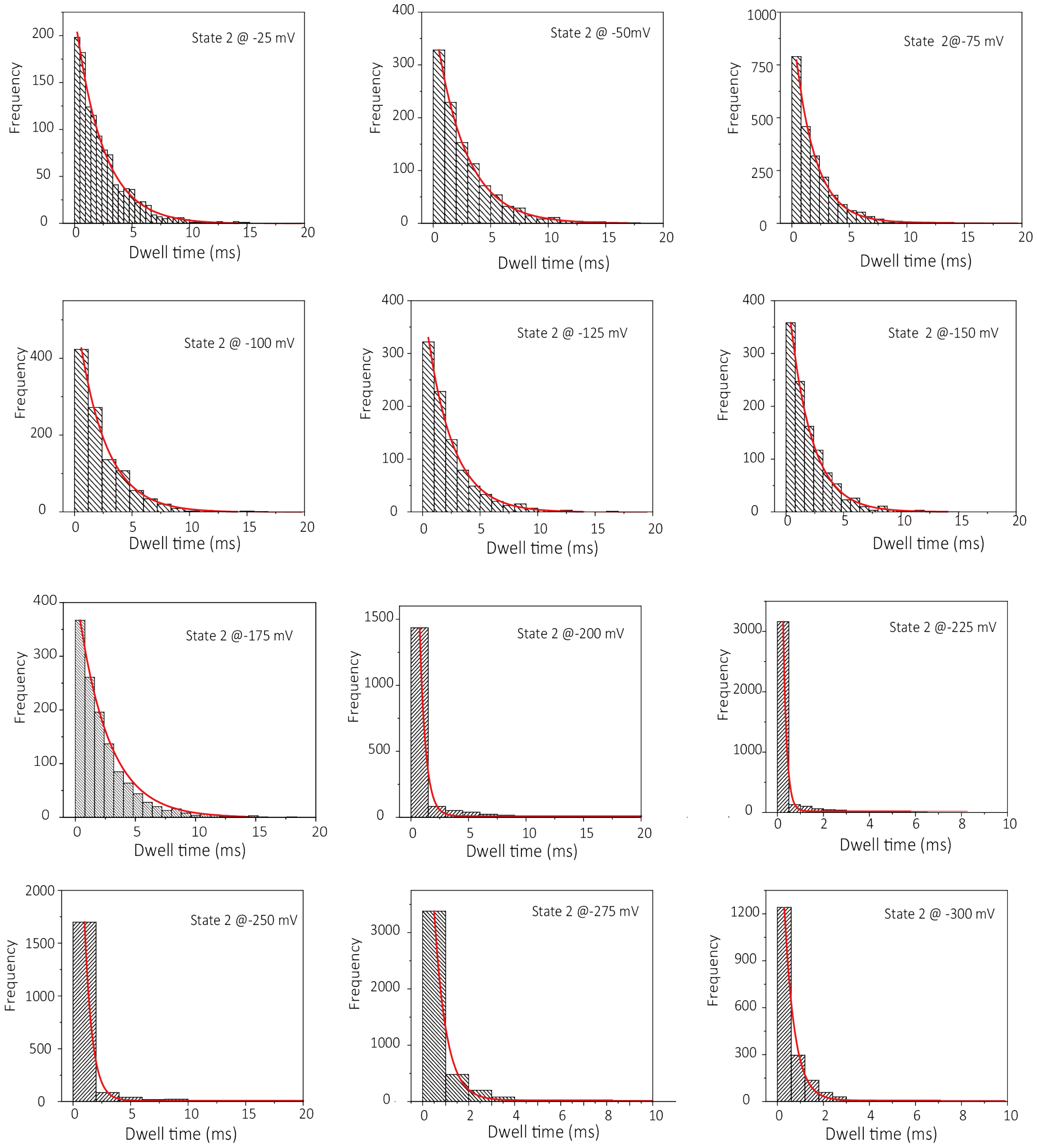

Supplementary Figure S18 Dwell time histograms for conductance state 2 for the streptavidin-coupled QMT probe in the different bias. The solid red line corresponds to monoexponential fits from which the lifetime constant τ_{s2} has been calculated. Transition lifetimes were obtained by a monoexponential fit of the dwell time histogram.

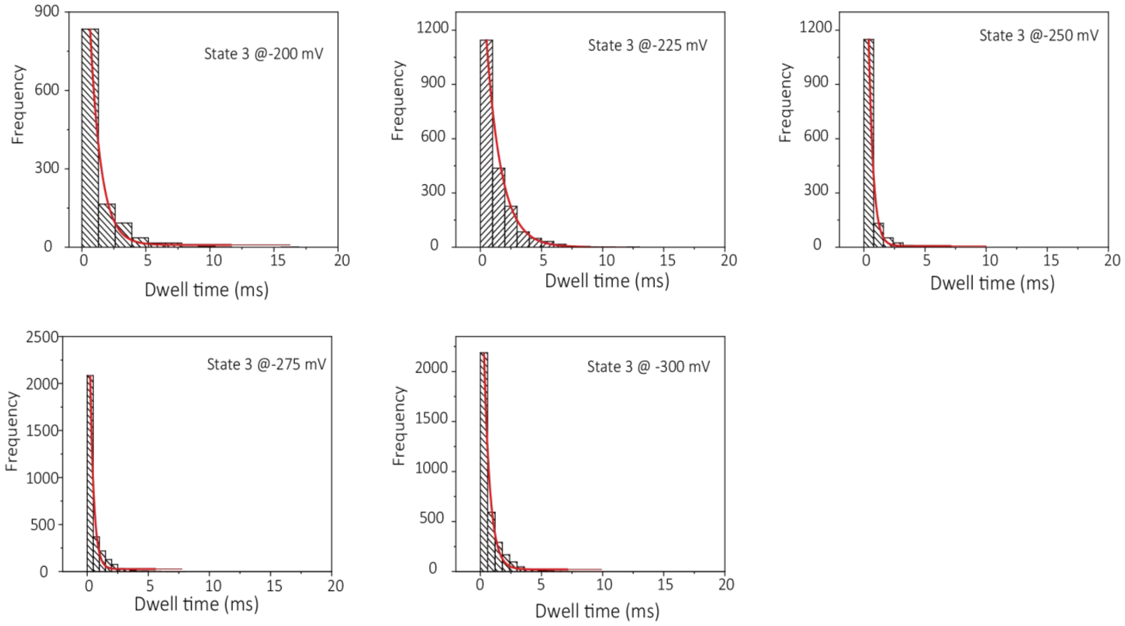

SupplementaryFigure S19 Dwell time histograms for conductance state 3 for the streptavidin-coupled QMT probe in the different bias. The solid red line corresponds to monoexponential fits from which the lifetime constant τ_{s3} has been calculated. Transition lifetimes were obtained by a monoexponential fit of the dwell time histogram.

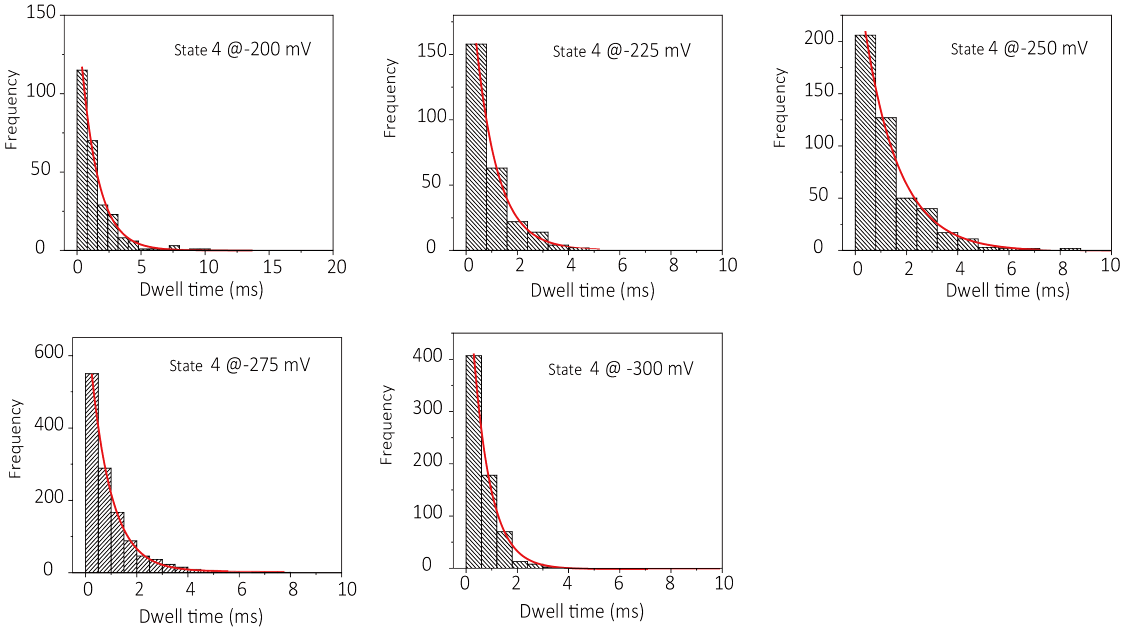

Supplementary Figure S20 Dwell time histograms for conductance state 4 for the streptavidin-coupled QMT probe in the different bias. The solid red line corresponds to monoexponential fits from which the lifetime constant τ_{s4} has been

calculated. Transition lifetimes were obtained by a monoexponential fit of the dwell time histogram.

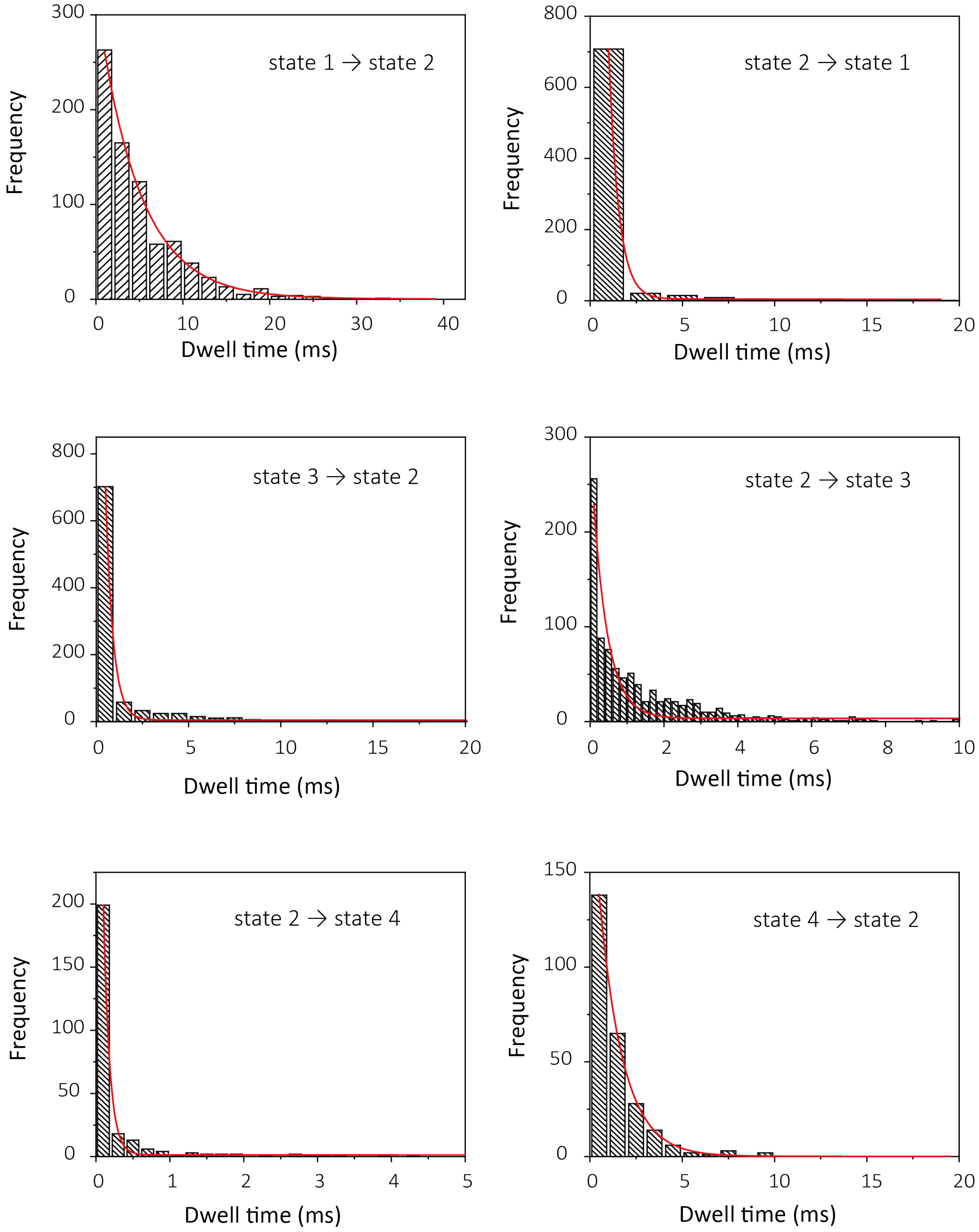

Supplementary Figure S21 Transition statistics analysis between each state for the current-time trace measured at -200 mV. Plots show the time intervals of three reversible transitions of state 1 ↔ state 2, state 3↔ state 2, and state 2 ↔ state 4. Transition lifetimes were obtained by a monoexponential fit of the dwell time histogram.

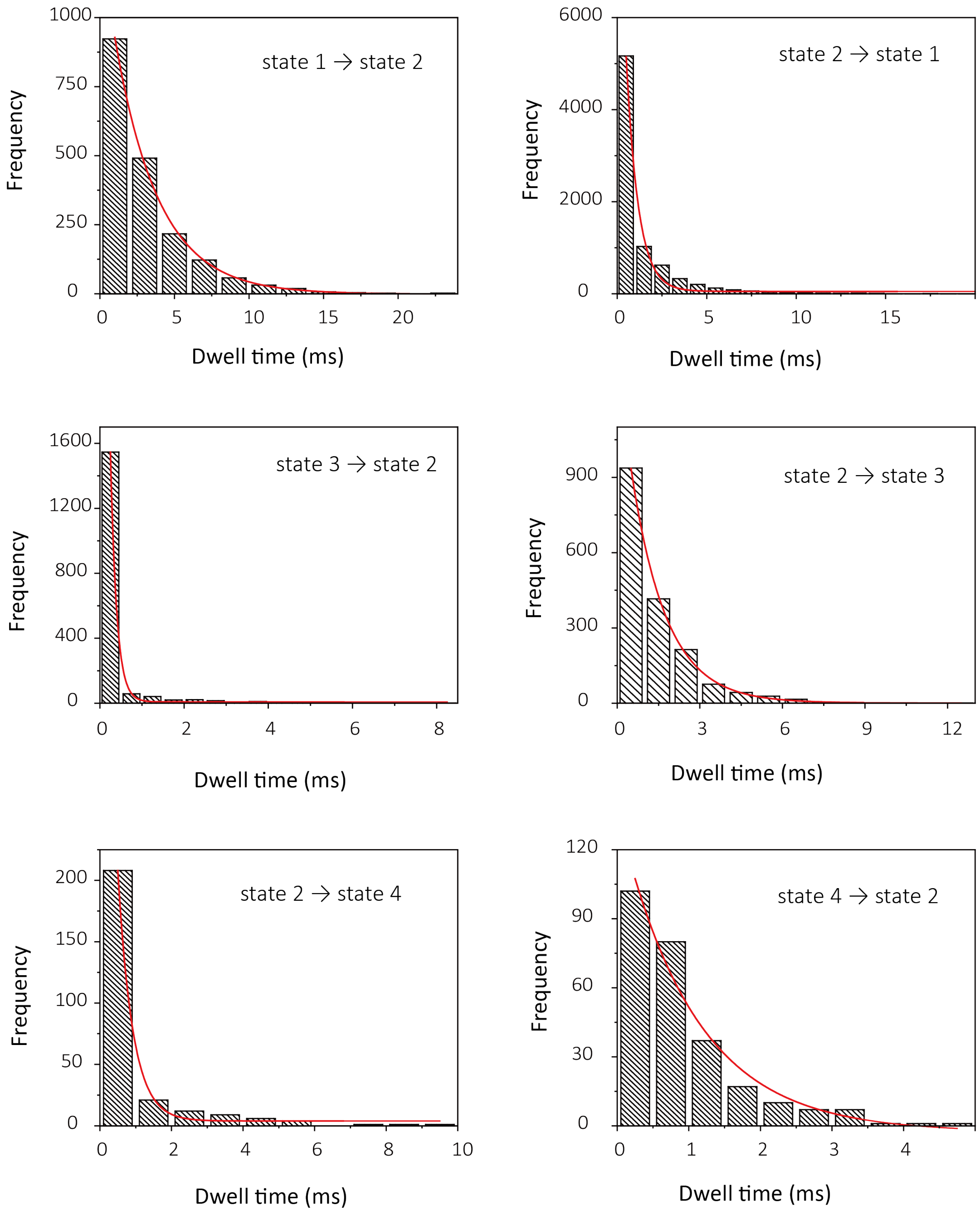

Supplementary Figure S22 Transition statistics analysis between each state for the current-time trace measured at -225 mV. Plots show the time intervals of three reversible transitions of state 1 ↔ state 2, state 3↔ state 2, and state 2 ↔ state 4. Transition lifetimes were obtained by a monoexponential fit of the dwell time histogram.

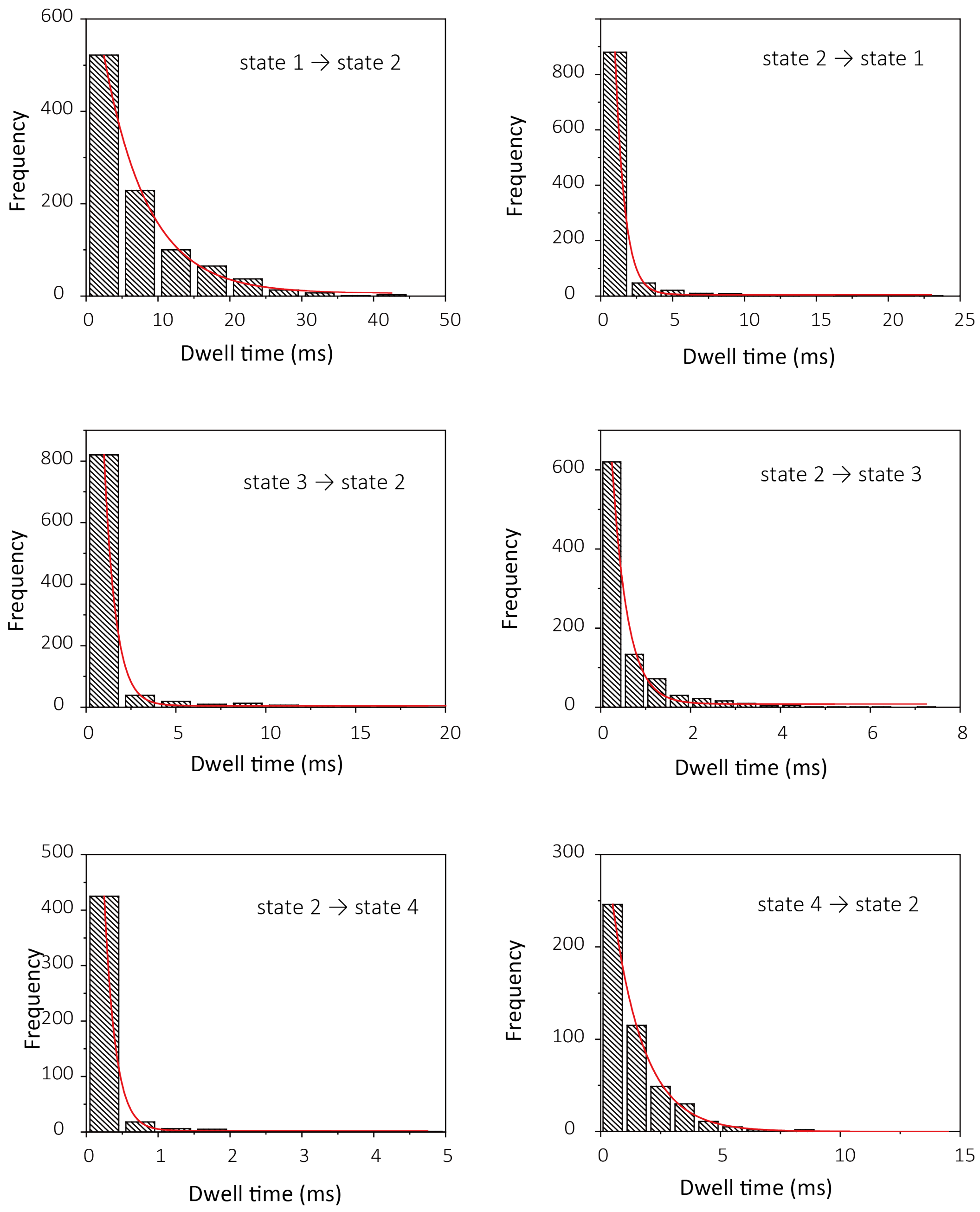

Supplementary Figure S23 Transition statistics analysis between each state for the current-time trace measured at -250 mV. Plots show the time intervals of three reversible transitions of state 1 ↔ state 2, state 3↔ state 2, and state 2 ↔ state 4. Transition lifetimes were obtained by a monoexponential fit of the dwell time histogram.

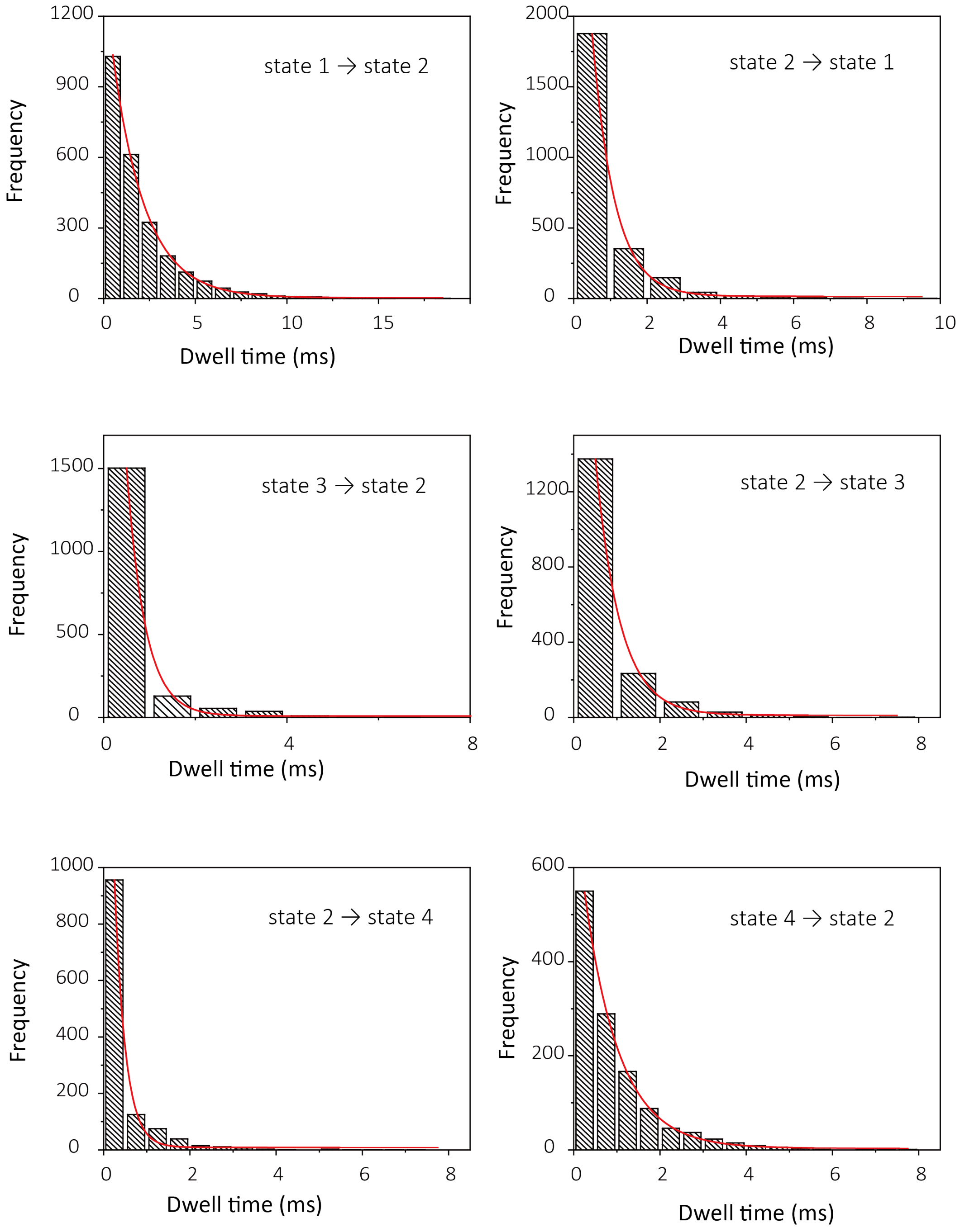

Supplementary Figure S24 Transition statistics analysis between each states for the current-time trace measured at -275 mV. Plots show the time intervals of three reversible transitions of state 1 ↔ state 2, state 3↔ state 2, and state 2 ↔ state 4. Transition lifetimes were obtained by a monoexponential fit of the dwell time histogram.

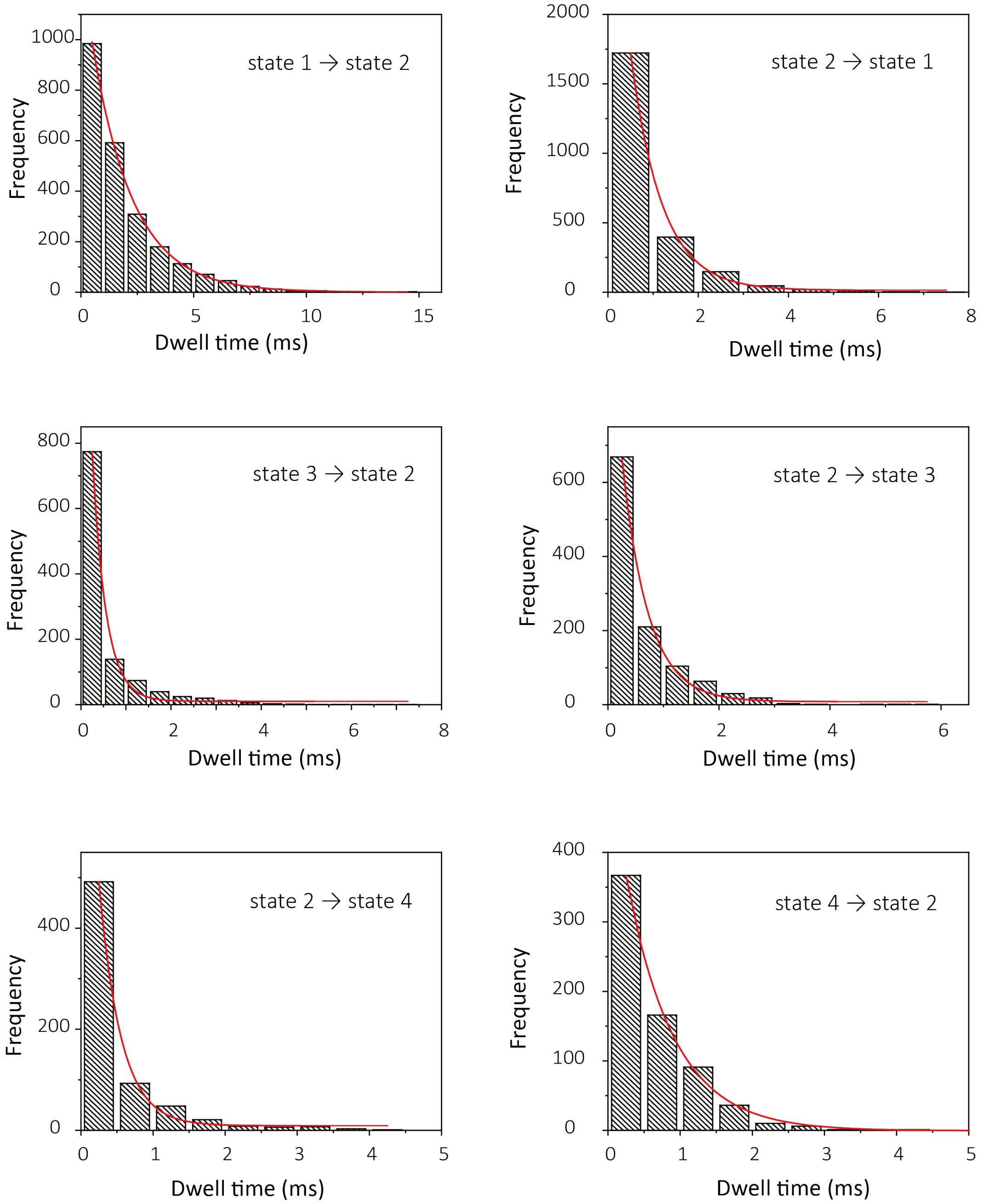

Supplementary Figure S25 Transition statistics analysis between each state for the current-time trace measured at -300 mV. Plots show the time intervals of three reversible transitions of state 1 ↔ state 2, state 3↔ state 2, and state 2 ↔ state 4. Transition lifetimes were obtained by a monoexponential fit of the dwell time histogram.

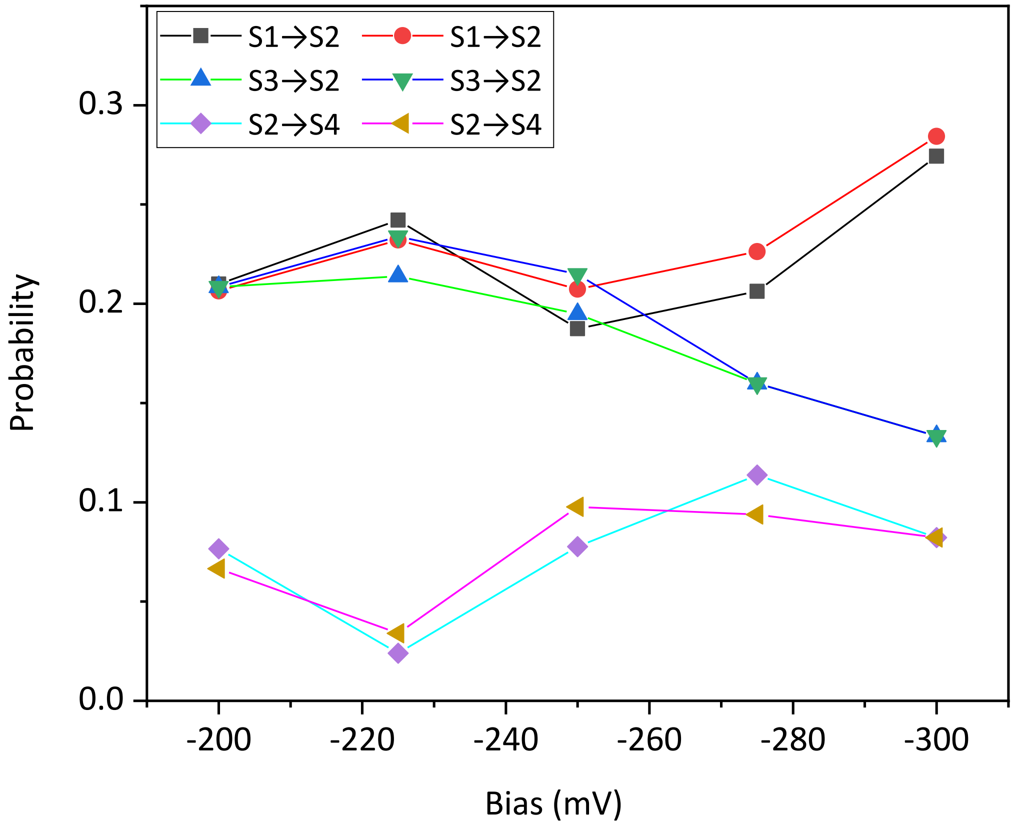

Supplementary Figure S26 Plots of transition probability for each state as a function of bias.

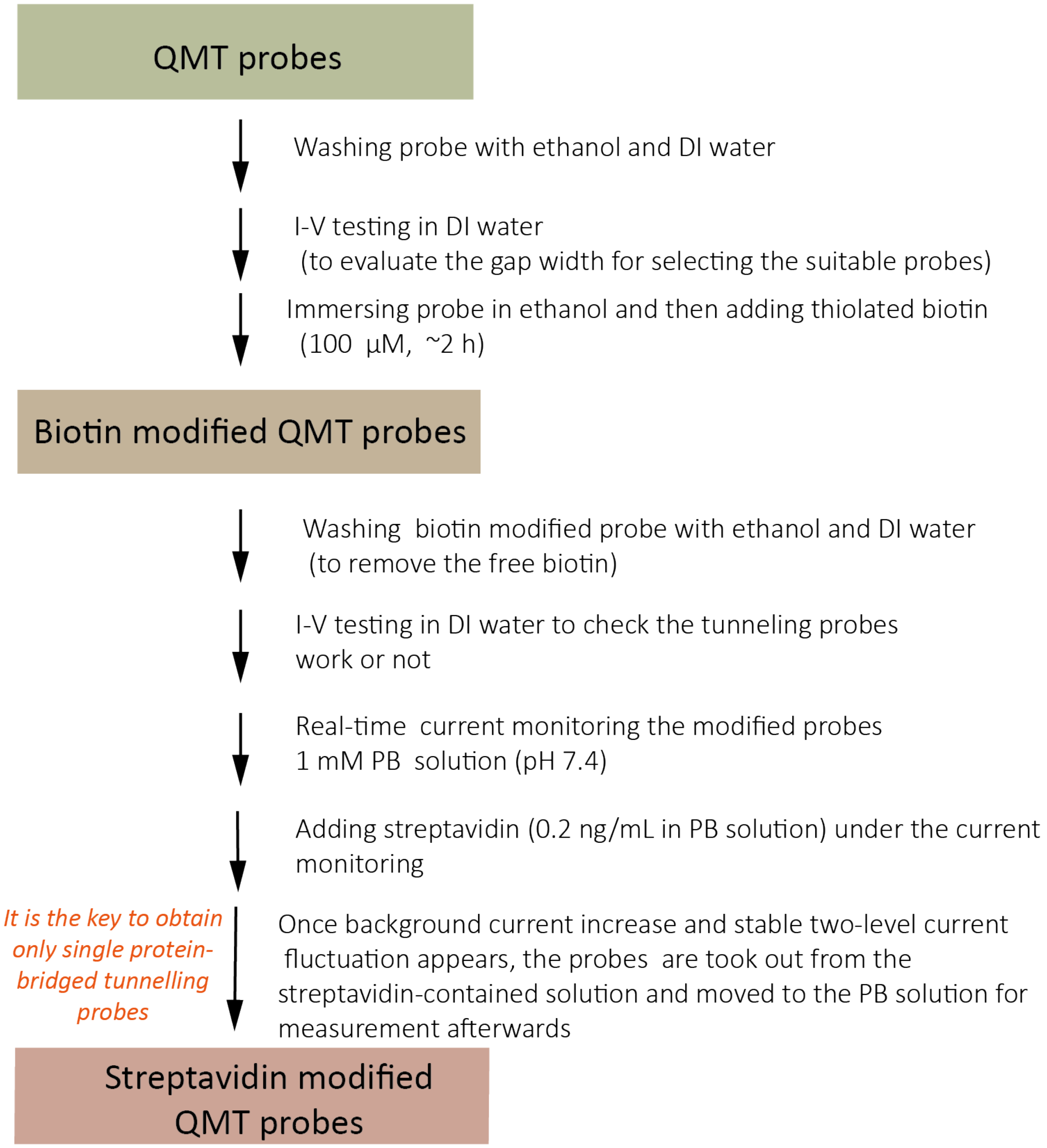

Supplementary Figure S27 Workflow of the fabrication of streptavidin-modified QMT probes.

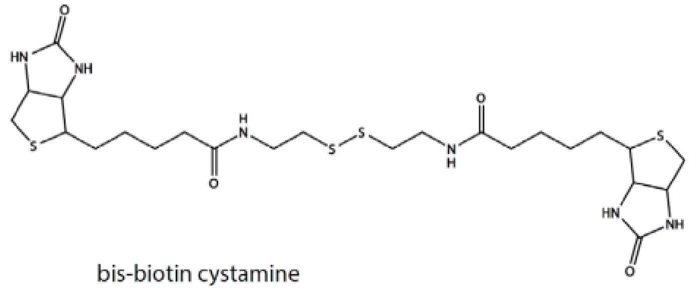

bis-biotin cystamine

Supplementary Figure S28 The structure of N,N'-bisbiotinyl-cystamine (MW:604, size <1 nm)

Bias (mV)	State 1			State 2			State 3			State 4			k (ms^{-1})
	G_{s1} (nS)	τ_{s1}(ms)	γ_{s1}	G_{s2}(nS)	τ_{s2}(ms)	γ_{s2}	G_{s3}(nS)	τ_{s3}(ms)	γ_{s3}	G_{s4}(nS)	τ_{s4}(ms)	γ_{S4}	
-25	17.1±1.0	5.00±0.27	72.5%	23.3±1.7	2.44±0.08	24.4%	-	-	-	-	-	-	0.27
-50	19.1±1.3	6.05±0.22	70.4%	27.5±1.1	2.64±0.04	26.4%	-	-	-	-	-	-	0.23
-75	17.8±0.9	3.99±0.18	72.3%	21.7±1.2	1.79±0.04	17.9%	-	-	-	-	-	-	0.35
-100	13.4±0.6	6.74±0.13	74.9%	20.0±1.6	2.40±0.09	24.0%	-	-	-	-	-	-	0.22
-125	15.6±0.8	6.23±0.2	74.7%	23.0±1.4	2.26±0.07	22.6%	-	-	-	-	-	-	0.24
-150	16.5±0.8	7.33±0.44	76.8%	24.9±1.2	1.94±0.05	19.4%	-	-	-	-	-	-	0.22
-175	18.1±0.6	4.48±0.08	65.8%	27.4±0.9	2.32±0.04	23.2%	-	-	-	-	-	-	0.30
-200	19.8±0.8	4.81±0.25	58.8%	23.9±1.1	0.52±0.04	5.2%	29.9±1.2	0.85±0.06	16.0%	33.2±1.8	0.85±0.06	5.6%	0.57
-225	22.3±1.2	2.79±0.1	57.9%	26.6±1.0	0.15±0.01	1.5%	32.8±1.5	1.23±0.06	10.7%	36.7±1.5	0.86±0.04	4.6%	0.28
-250	22.5±0.9	2.31±0.2	65.3%	28.2±1.4	0.66±0.03	6.6%	34.8±1.1	0.37±0.02	10.9%	38.7±2.1	1.35±0.09	6.5%	0.85
-275	27.1±0.9	1.78±0.03	49.3%	32.6±1.2	0.52±0.03	5.2%	38.7±1.4	0.31±0.02	23.7%	43.3±1.4	0.81±0.01	11.8%	1.17
-300	29.9±0.9	1.81±0.03	51.0%	35.4±1.9	0.50±0.03	5.0%	42.0±1.6	0.45±0.02	25.0%	46.8±1.9	0.68±0.02	17.3%	1.16

Supplementary Table S1 Summary of the conductance G, lifetime τ, occupancy γ, and conductance switching rate constant k at different bias.

Bias (mV)	state 1 → state 2			state 2 → state 1			state 3→state 2			state 2→state 3			state 2 → state 4			state 4 → state 2		
	$\tau_{s1 \rightarrow S2}$ (ms)	$k_{s1 \rightarrow S2}$ (ms^{-1})	p	$\tau_{s2 \rightarrow S1}$ (ms)	$k_{s2 \rightarrow S1}$ (ms^{-1})	p	$\tau_{s3\text{-}S2}$ (ms)	$k_{s3 \rightarrow S2}$ (ms^{-1})	p	$\tau_{s2 \rightarrow S3}$ (ms)	$k_{s2 \rightarrow S3}$ (ms^{-1})	p	$\tau_{s2 \rightarrow S4}$ (ms)	$k_{s2 \rightarrow S4}$ (ms^{-1})	p	$\tau_{s4 \rightarrow s2}$ (ms^{-1})	$k_{s4 \rightarrow s2}$ (ms^{-1})	p
-200	0.44±1.95	2.27±0.51	0.21	4.51±1.78	0.22±0.56	0.21	4.62±1.46	0.22±0.68	0.21	3.39±0.98	0.29±1.02	0.21	1.36±0.46	0.74±2.17	0.08	0.16±0.24	6.25±4.17	0.07
-225	6.92±4.20	0.14±0.24	0.24	12.11±4.50	0.08±0.22	0.23	9.17±5.09	0.11±0.20	0.21	1.45±3.24	0.69±0.31	0.23	2.32±1.03	0.43±0.97	0.02	0.36±0.47	2.78±2.13	0.03
-250	6.04±4.52	0.17±0.22	0.19	4.63±1.96	0.22±0.51	0.21	4.69 1.71	0.21±0.58	0.19	8.07±3.73	0.12±0.27	0.21	2.01±0.74	0.50±1.35	0.09	0.06±0.66	16.67±1.52	0.10
-275	3.22±2.37	0.31±0.42	0.20	14.79±10.41	0.07±0.10	0.23	9.00±5.365	0.11±0.19	0.16	12.05±7.58	0.08±0.13	0.16	8.64±4.74	0.12±0.21	0.11	3.42±1.55	0.29±0.65	0.11
-300	1.40±3.56	0.71±0.28	0.27	14.57±9.68	0.07±0.10	0.28	10.70±4.57	0.09±0.22	0.13	8.36±5.61	0.12±0.18	0.13	7.02±3.15	0.14±0.32	0.08	6.18±0.94	0.10±1.06	0.08

Supplementary Table S2 Summary of the lifetime τ, rate constant k and probability (p) for each conductance transition obtained from the statistic analysis in Supplementary Figure S22-S26.

We further analyzed the transitions probabilities for each conductance state at different bias conditions. At the low bias <200 mV, the dominant transition is between S1 and S2. As the bias increased to the higher bias range (-200 mV~ -300 mV), we found that three main reversible transitions (i.e., S1↔S2, S3↔S2, S2↔S4) existed, whereas other pathways were barely observed (<2% of the total transitions). By taking the bias of -300 mV as an example, the dwell times of all the most pronounced six-transition processes were determined by analyzing idealized current traces with Hidden Markov modeling and further fitted by a single exponential decay, as shown in Supplementary Figs. S21-S25. The average lifetime were obtained as follows: $\tau_{s1 \rightarrow S2}$ = 1.40 ± 3.56 ms, $\tau_{s2 \rightarrow s1}$ = 14.57 ± 9.68 ms, $\tau_{s3 \rightarrow s2}$ =10.70 ± 4.57 ms, $\tau_{s2 \rightarrow s3}$ = 8.36 ± 5.61 ms, $\tau_{s2 \rightarrow s4}$ = 7.02± 3.15 ms, and $\tau_{s4 \rightarrow s2}$ = 6.18 ± 0.94 ms, respectively. On the basis of the dwell time calculation, the rate constant corresponding to the transition were derived to be $k_{s1 \rightarrow S2}$ = 0.71 ± 0.28 ms^{-1}, $k_{s3 \rightarrow s2}$ = 0.09 ± 0.22 ms^{-1}, $k_{s2 \rightarrow s4}$ = 0.14±0.32 ms^{-1}, $k_{s2 \rightarrow S1}$ = 0.07 ± 0.10 ms^{-1}, $k_{s2 \rightarrow s3}$ = 0.12 ± 0.18 ms^{-1}, and $k_{s4 \rightarrow s2}$ = 0.10 ± 1.06 ms^{-1}, respectively. Similar analyses for other biases were performed by using the same process, and more data are shown in Table S2. These results suggest that the protein conductance switching with bias should mainly comply with the sequence of S1↔S2↔S3↔S4, and the transition between S1 and S2 are predominant under different bias conditions.
